# Development and application of the direct mycobacterial growth inhibition assay: a systematic review

**DOI:** 10.3389/fimmu.2024.1355983

**Published:** 2024-02-06

**Authors:** Hannah Painter, Eli Harriss, Helen A. Fletcher, Helen McShane, Rachel Tanner

**Affiliations:** ^1^ Department of Infection Biology, Faculty of Infectious and Tropical Diseases, London School of Hygiene and Tropical Medicine, London, United Kingdom; ^2^ Bodleian Health Care Libraries, University of Oxford, Oxford, United Kingdom; ^3^ Nuffield Department of Medicine, Jenner Institute, University of Oxford, Oxford, United Kingdom; ^4^ Department of Biology, University of Oxford, Oxford, United Kingdom

**Keywords:** mycobacterial growth inhibition assay, MGIA, tuberculosis, vaccines, BCG

## Abstract

**Introduction:**

First described by Wallis et al. in 2001 for the assessment of TB drugs, the direct mycobacterial growth inhibition assay (MGIA) offers a tractable *ex vivo* tool measuring the combined influences of host immunity, strain virulence and intervention effects. Over the past 13 years, we have led efforts to adapt the direct MGIA for the assessment of TB vaccines including optimisation, harmonisation and validation of BCG vaccine-induced responses as a benchmark, as well as assay transfer to institutes worldwide.

**Methods:**

We have performed a systematic review on the primary published literature describing the development and applications of the direct MGIA from 2001 to June 2023 in accordance with the PRISMA reporting guidelines.

**Results:**

We describe 63 studies in which the direct MGIA has been applied across species for the evaluation of TB drugs and novel TB vaccine candidates, the study of clinical cohorts including those with comorbidities, and to further understanding of potential immune correlates of protection from TB. We provide a comprehensive update on progress of the assay since its conception and critically evaluate current findings and evidence supporting its utility, highlighting priorities for future directions.

**Discussion:**

While further standardisation and validation work is required, significant advancements have been made in the past two decades. The direct MGIA provides a potentially valuable tool for the early evaluation of TB drug and vaccine candidates, clinical cohorts, and immune mechanisms of mycobacterial control.

**Systematic review registration:**

https://www.crd.york.ac.uk/prospero/, identifier CRD42023423491.

## 1 Introduction

Functional *in vitro/ex vivo* assays such as growth/invasion inhibition assays (GIAs) aim to provide a surrogate measure of treatment or vaccine efficacy, reflecting the combined influences of host immunity, strain virulence and intervention effects. They have been applied with some degree of success to a range of disease models including HIV, malaria and meningitis (1–3). Given the lack of validated immune biomarkers or correlates of protection/treatment efficacy in the tuberculosis (TB) field, a successful mycobacterial GIA (MGIA) would be a particularly valuable complementary tool. Such an assay could permit low-cost high-throughput testing and down-selection of novel drug and vaccine candidates at an early stage of development, thus expediting the race to control the global TB epidemic. Furthermore, in line with the 3Rs principles of replacement, reduction and refinement of animal use in scientific research, MGIAs offer an alternative to *in vivo* ‘challenge’ experiments in which animals are infected with virulent *M.tb* in early screening of drug or vaccine efficacy (4).

A number of MGIAs have been previously described in the literature, including the use of reporter strains in whole blood (5, 6), primary or secondary lymphocyte-monocyte co-cultures (7, 8), bone marrow macrophage-splenocyte co-cultures (9, 10) and cattle peripheral blood mononuclear cells (PBMC) (11, 12) among others. These have been comprehensively reviewed elsewhere (13). However, in most cases, limited follow-up work was conducted to qualify and validate an MGIA that could be transferred across laboratories using a standardised reproducible method. From 2010-2014, an international consortium supported by Aeras TB Vaccine Foundation and the US Food and Drug Administration (FDA) aimed to define an MGIA that could be applied across clinical trials of TB vaccine candidates and aid in the identification of immune correlates of protection (14). Ultimately, an assay based on the methods of Wallis et al. was selected for further development due to its proven ability to detect anti-mycobacterial activity, relative simplicity, and use of standardised reagents and equipment aiding transferability (14).

In the first iteration of what is now widely referred to as the ‘direct MGIA’, Wallis et al. described their bactericidal assay as an adaptation of Schlichter and MacLean’s methods for monitoring infective endocarditis therapy (15). In brief, mycobacteria were inoculated directly into a patient blood sample followed by a 72 hour co-culture period before quantification of remaining bacilli (relative to a control sample) using the BACTEC MGIT system (16). This quantification system, developed as a diagnostic tool, has several advantages over traditional CFU-based methods, utilising an oxygen-quenched fluorochrome as a detection method making it sensitive to bacterial metabolism and growth as well as viable colonies. Furthermore, the BACTEC MGIT is unaffected by clumping and does not require serial dilutions or subjective manual counting, providing an accurate computer-generated read-out based on validated technology (14). Following the success of the consortium and the promising preliminary data generated (14), the direct MGIA has since been further optimised, standardised, harmonised and applied by several additional laboratories.

We have conducted a systematic review with the aim of providing a comprehensive update on the published progress and applications of the direct MGIA. We evaluate the evidence supporting this assay as a useful tool for the evaluation of TB drug and vaccine efficacy as well as the understanding of clinical *M.tb* infection, TB disease, coinfections and associated immune mechanisms of control.

## 2 Methods

### 2.1 Search strategy and selection criteria

A systematic review was performed of studies pertaining to the development and application of the direct MGIA, in accordance with guidance from the Preferred Reporting Items for Systematic reviews and Meta-Analyses (PRISMA) (17). The protocol was published (https://www.crd.york.ac.uk/PROSPEROFILES/423491_PROTOCOL_20230507.pdf) and the review was registered on PROSPERO prior to commencing (ID: CRD42023423491). An information specialist (EH) searched PubMed, Ovid Embase and Scopus on 22/05/2023 for relevant records published from 2001 to the search date. The search strategies used text words and relevant indexing terms where applicable for the bibliographic databases; the full search strategies are provided in **Supplementary Table 1**. All references were exported to EndNote 20 (Thomson Reuters, New York, NY), and the records were de-duplicated using the method for EndNote developed by Falconer (18). Search results were imported into Rayyan (Qatar Computing Research Institute) (19) for independent screening by two researchers (HP and RT).

The following inclusion criteria were applied: i) published between 2001 (first reported description of the direct MGIA) and May 22^nd^ 2023 (date of the first search); ii) published in a peer-reviewed journal; iii) published in the English language; and iv) pertaining wholly or partially to the direct MGIA (defined as the co-culture of mycobacteria with primary whole blood or primary cells followed by quantification using the BACTEC MGIT system), as applied to any species and in relation to any cohort or research question. Studies were examined for relevance in two rounds of screening. In the first round, titles and abstracts were assessed and those that were irrelevant or duplicates were excluded. Ambiguous cases were examined in a second round, where the full texts were accessed, and exclusions were made largely on the basis of being: i) about an MGIA other than the direct MGIA, including the use of cell lines or alternative mycobacterial quantification methods that were not BACTEC MGIT; ii) a conference abstract, a review, or otherwise not peer-reviewed; or iii) inaccessible via the host institution or otherwise unable to locate. Finally, reference lists were examined for missed citations due to uncommon terms.

### 2.2 Data collection and synthesis of findings

In addition to recording the title, year, journal and authors, data was collected from eligible papers (were available) on: i) study groups analysed using the direct MGIA (species, cohorts, sample sizes, interventions); ii) direct MGIA methods (sample type, cell input/volume, mycobacterial inoculum type and quantity, co-culture volume and time period); and iii) other immune parameters measured and whether they were assessed for correlation with control of mycobacterial growth. Data was synthesised and organised by application i.e., study of drugs, vaccines or clinical cohorts; and within application by species (or disease state in the case of clinical cohorts). Data pertaining to immune correlates from all studies was organised into innate and adaptive arms and then by immune parameter (e.g., cellular subset). Quality of the included studies (considering the direct MGIA aspects only) was evaluated using a strategy adapted from the Quality Assessment of Controlled Intervention Studies tool developed by the National Heart, Lung and Blood Institute (NHLBI) (20) and the Quality Assessment Tool for *in vitro* Studies (QUIN) developed by Sheth et al. (21) to ensure relevance to the nature of the review and inclusion criteria. Studies were rated according to 12 criteria concerning: clarity of study question or objective, pre-specification and description of study population(s), sample size, description and delivery of intervention(s), method of randomisation, control group(s), timeframe, blinding, replicates, description of methods, outcome measures, and statistical analysis. Possible responses were yes (1), no (-1), not reported (0), or not applicable (0). Scores were combined to give a total score and assign a quality category as follows: 0-2 = poor, 3-5 = fair, 6-8 = good, 9-10 = very good, 11-12 = excellent.

## 3 Results

**Figure 1:**
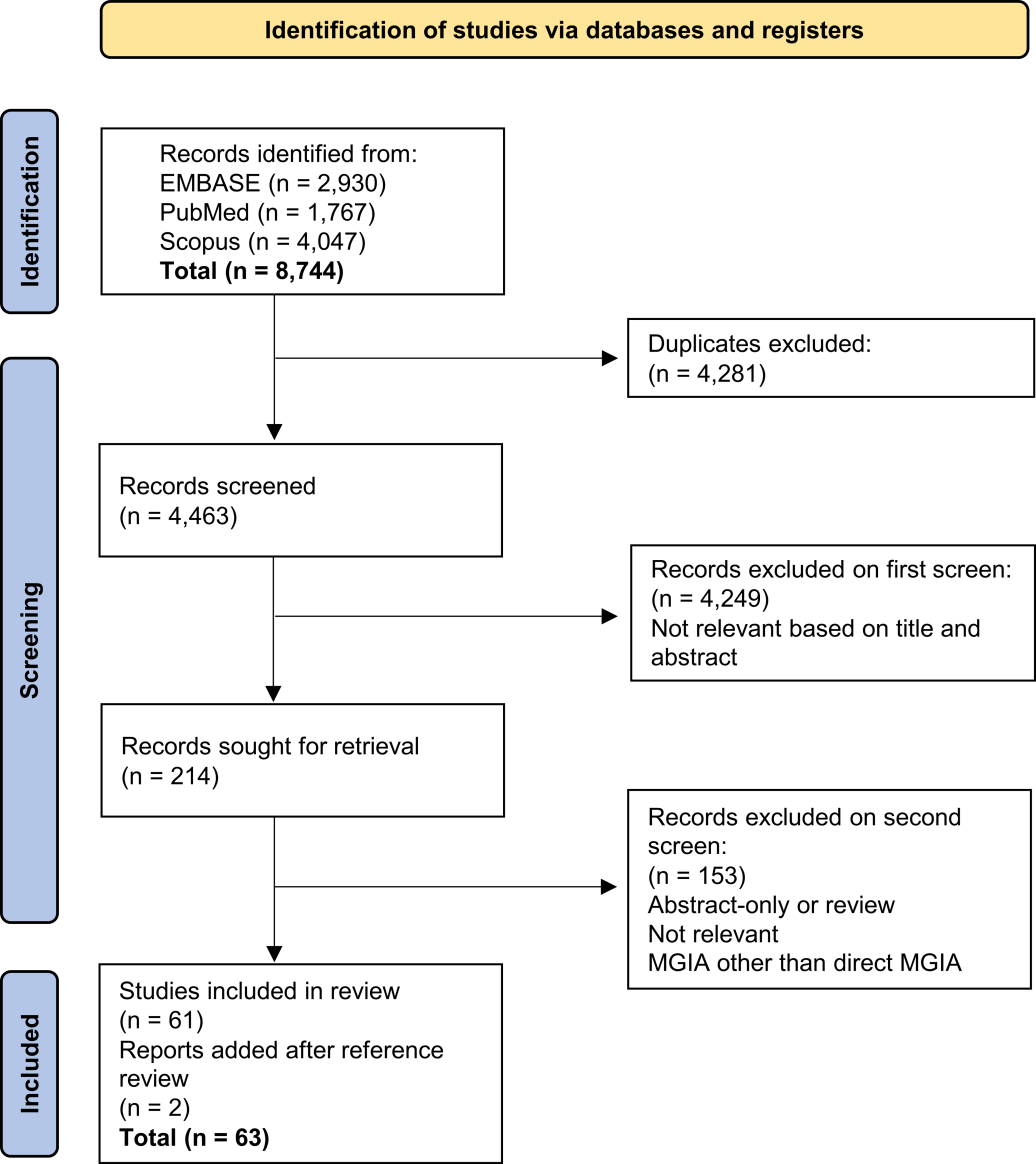
Flow diagram of search process and publication selection.

8,744 records were initially returned. Following removal of duplicates and two rounds of screening, 61 records were included in the systematic review and a further two were added from reference screening (**Figure 1**). The predominant reason for exclusion was the measurement of bactericidal activity using methods such as minimum inhibitory concentration, minimum bactericidal concentration or early bactericidal activity but not the direct MGIA. Furthermore, a portion of studies measured control of mycobacterial growth but in cell lines rather than primary cells, or used primary cells but a different co-culture method and/or a different mycobacterial quantification method such as traditional 7H11 agar plating or measures of luminescence. Such assays were not considered to be the direct MGIA and were therefore beyond the scope of this review.

**Figure 2:**
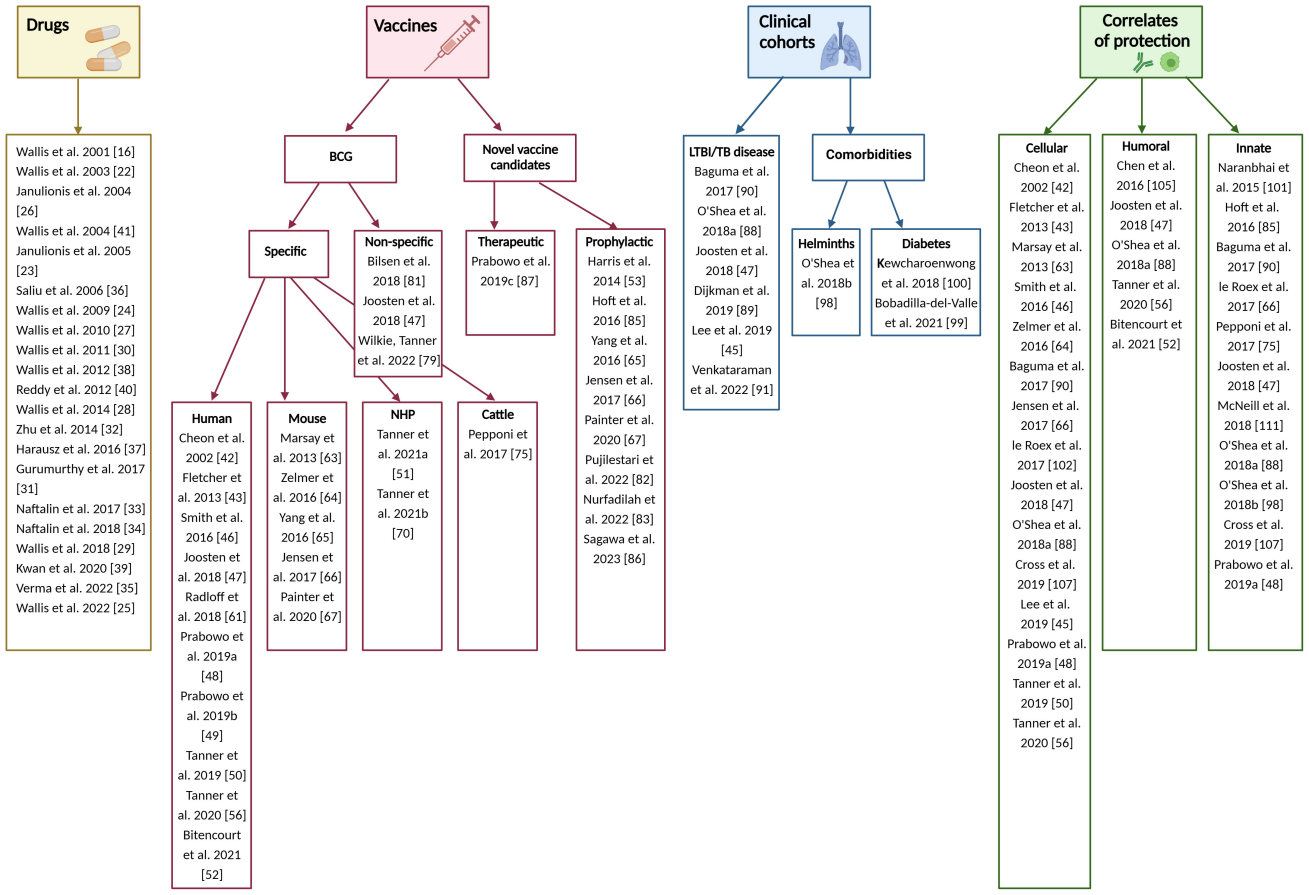
Organisation of included publications. Created in BioRender.com.

**Figure 3:**
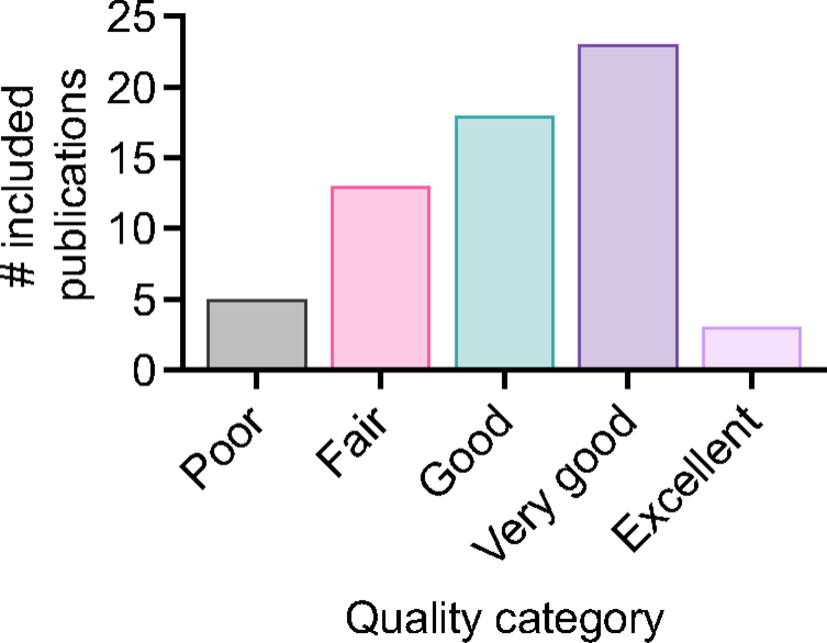
Distribution of quality assessment scores for included studies.

For included studies, the distribution of publications across different applications of the MGIA is summarised in **Figure 2**. 22 studies pertained to the testing of TB drugs, and 29 to TB vaccines including nine that evaluated novel TB vaccine candidates. There were 10 studies in clinical cohorts including 3 considering the effects of comorbidities, and 25 studies that explored immune correlates of mycobacterial growth control. Some studies were included under more than one category; for example they may have explored correlates of mycobacterial growth control as well as evaluated a TB vaccine candidate. The data collected from these studies is summarised in **Supplementary Table 2**. Regarding study quality there was a range of outcomes, with 71% of studies rated ‘good’ or above. Three studies received an ‘excellent’ rating, 23 were considered ‘very good’, 18 were rated ‘good’, 13 received a ‘fair’ rating, and five were considered ‘poor’ (**Figure 3**). Details of the quality assessment are provided in **Supplementary Table 3**.

### 3.1 Development of the direct MGIA for the evaluation of TB drugs

In the first direct MGIA study described by Wallis et al., blood was collected before, and at intervals after, administration of eight TB drug regimens, and co-cultured with the attenuated laboratory strain *M.tb* H37Ra or a drug-sensitive clinical isolate. After 72 hours, cells were lysed and remaining bacilli quantified using the BACTEC MGIT system. Mycobacterial survival was determined by comparing the CFU for experimental and control cultures (16). It was concluded that the effects observed in the MGIA correlated well with sterilising activity observed *in vivo* during therapy, and that the model showed promise for the evaluation of new drugs and monitoring of individual patient therapies (16). In 2003, the authors reported a larger study of bactericidal activity in whole blood from 30 TB patients before, during and after completion of daily treatment with isoniazid, rifampicin, ethambutol and pyrazinamide for 60 days, followed by isoniazid and rifampicin for 120 days (22).

Further reports from the same group describe the assay applied to whole blood taken from different volunteer groups to study the survival and replication of clinical *M.tb* isolates or laboratory strains in the context of host innate immunity (23), and the control of different *M.tb* strains in blood from cured TB patients and tuberculin-negative volunteers (24). With respect to the screening of various TB drug therapies, Wallis and others have also reported on the effect of host-directed TB therapies (25), the activity of orally-administered clofazimine, PNU-100480 alone or with bedaquiline plus rifabutin or rifampicin (26–30), activity of faropenem with and without rifampicin (31), activity of sutezolid (PNU-100480) and its major metabolite (32), coadministration of allopurinol with pyrazinamide (33), and adjunctive use of celecoxib with anti-tuberculosis drugs (34).

In an alternative complementary approach, spiking healthy whole blood co-cultures with therapeutic agents has also allowed the rapid evaluation of drugs including the effects of increasing concentration of rifampicin on different *M.tb* lineages (35), the influence of TNF blockers on mycobacterial immunity (36), bactericidal activity of nitazoxanide and tozoxanide against drug-tolerant *M.tb* (37), regimens for XDR-TB containing PNU-100480, TMC207, PA-824, SQ109 or pyrazinamide (38), gene expression responses to anti-tuberculous drugs (39), and the interaction between SQ109 and PNU-100480 in killing *M.tb* (40). Wallis et al. also illustrated the importance of assessing TB drug activity in the context of host immunity, showing that the effect of ofloxacin is markedly reduced in whole blood from TB patients (in whom immune antimycobacterial mechanisms are activated) compared with that of healthy controls (41). In most cases the assay conditions used were consistent with 0.3ml of whole blood in a total co-culture volume of 0.6ml and a mycobacterial input predicted to be positive in 4.5 to 5.5 days, although the input CFU was often undefined and there were some minor deviations such as 24 or 96 hour co-culture periods.

### 3.2 Adaptation of the direct MGIA for the assessment of TB vaccines

#### 3.2.1 Specific effects of BCG vaccination

##### 3.2.1.1 Human direct MGIA

The first application of the Wallis bactericidal assay to the study of vaccine-induced enhancement of mycobacterial control was reported in 2002, when the same group assessed *M. bovis* BCG growth in whole blood from 10 healthy US adults taken before and after primary BCG vaccination and revaccination 6 months later (42). Overall, there was a significant enhancement in control following BCG revaccination but not primary vaccination using both 72 and 96 hour co-culture periods (42).

In 2013, we reported a further study of primary and secondary BCG vaccination in healthy UK volunteers, assessing responses in a similar iteration of the direct MGIA using either whole blood and a novel adaptation using cryopreserved PBMC (43). No differences were detected over time in either group using the whole blood MGIA, but in PBMC, a significant improvement in control of mycobacterial growth was observed at 8 weeks following primary vaccination but not revaccination with BCG (43). The assay conditions used were a BCG inoculum of ~150 CFU for 300µl of whole blood and ~600 CFU for 1x10^6^ PBMC (when exposed to antibiotics in the culture medium) or ~250 CFU for 1x10^6^ PBMC (when not exposed to antibiotics). For both assays, co-cultures were incubated in rotating 2ml screw-cap tubes; 10% pooled human AB serum was included in the PBMC cultures. Preliminary assessments of reproducibility confirmed that both assays had an inter-assay coefficient of variation (CV) of <50% (a limit of acceptable variation suggested by Tuomela et al. for the measurement of a bacterial target in a cell-based assay (44)). The whole blood assay was found to have higher overall variability, likely due to variations in mycobacterial input and week-to-week performance of the assay which must be run in real-time, while the PBMC assay could be batched (43). Consistent with this, Lee et al. reported excellent intra-assay (within-run precision or repeatability) and inter-assay (between-run) precision coefficients of variation (% CV) of 2.92% and 6.44%, respectively, for the PBMC MGIA performed on 16 occasions using cells from Korean volunteers (45).

Between 2016 and 2019, the direct MGIA using PBMC and 10% pooled human AB serum inoculated with BCG in rotating 2ml screw-cap tubes (henceforth referred to as the ‘in-tube’ assay) was applied by a further three laboratories to successfully detect a BCG-vaccine induced response. Smith et al. reported a study of healthy UK infants that either received, or did not receive, BCG vaccination at ~5 weeks of age. Vaccinated infants had a significantly improved capacity to control mycobacterial growth in the MGIA at 4 months post-vaccination compared with unvaccinated controls; a difference which had waned by 1 year (46). Joosten et al. described a study of healthy Dutch donors assessed before and at 4, 8, 12 and 52 weeks after BCG vaccination. When the pre-vaccination result was compared with the result at the peak post-vaccination response for each individual, there was significant improvement in mycobacterial control in the MGIA overall, albeit modest and transient (47). The following year, Prabowo et al. demonstrated significantly improved control of mycobacterial growth in PBMC taken from healthy UK adults who were historically BCG vaccinated compared with unvaccinated controls (48, 49). Interestingly, BCG-vaccinated females exhibited a superior capacity to control mycobacterial growth compared to males (48). These studies used a range of cell inputs (from 1x10^6^ to 3x10^6^ PBMC) and BCG inputs (from 100 to 862 CFU) which, together with the differing populations, may account for inconsistent findings (46–48).

**Figure 4:**
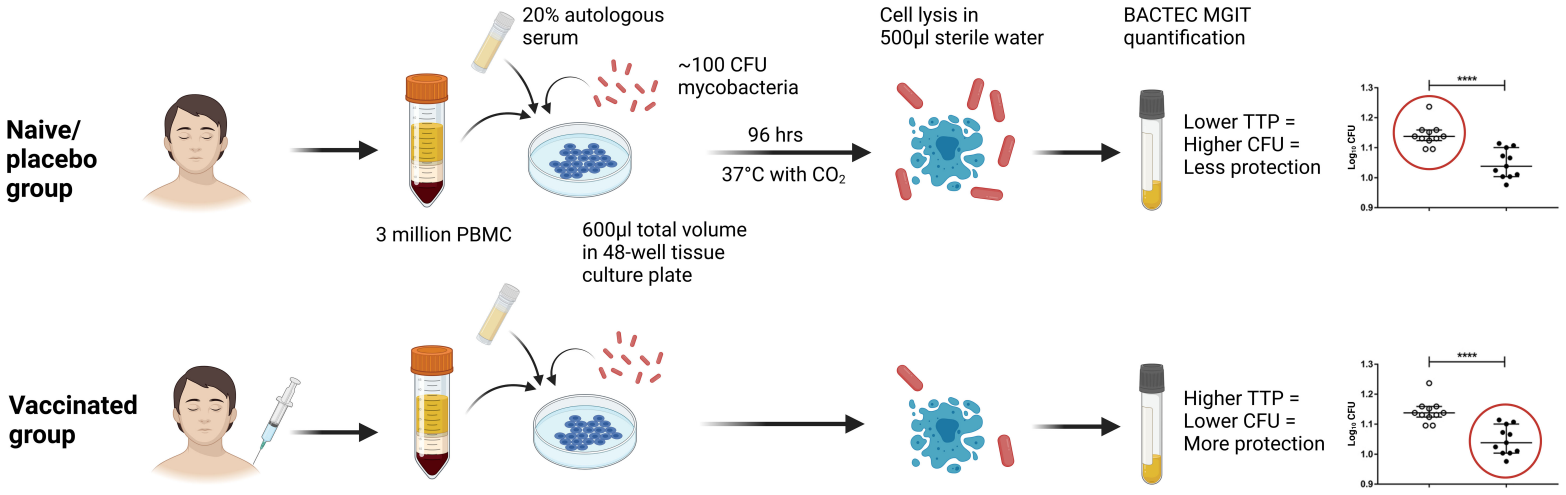
Schematic presentation of the direct PBMC MGIA method, summarising optimised assay conditions identified through the EURIPRED project (50). CFU, colony forming unit; TTP, time to positivity; MGIT, mycobacteria growth indicator tube. Created in BioRender.com.

To further optimise, harmonise and standardise the direct PBMC MGIA conditions, we led an MGIA sub-group of the FP7 European Research Infrastructures for Poverty Related Diseases (EURIPRED) consortium. In collaboration with three laboratories at different institutes across Europe, it was determined that intra-assay repeatability was improved by increasing the cell number and mycobacterial input (50). Furthermore, co-culturing in static 48-well plates compared with rotating 2ml tubes resulted in a 23% increase in cell viability and a 500-fold increase in IFN-γ production on average, as well as improved reproducibility between replicates, assay runs and sites. Applying these optimised conditions, we found intra-assay repeatability to be consistently <5% CV, intermediate (inter-assay) precision to be <20% CV, and inter-site reproducibility to be <30% CV (50). Using relevant clinical samples, the authors then demonstrated comparable results across two shared sample sets at three sites (50). In parallel, we performed substantial additional optimisation work exploring pre-culture conditions (such as length of cell rest and mycobacterial stock preparation), culture conditions (such as culture period and mixing), and post-culture processing (including cell lysis agents, supernatant removal and centrifugation steps) (51). Taken together, this supported the development of a standardised operating procedure (SOP) for the use of the direct PBMC MGIA (50), the conditions of which are summarised in **Figure 4**. In a later publication, we applied this protocol to demonstrate a significant BCG vaccine-induced MGIA response in two cohorts, one from a TB-endemic and one from a non-TB endemic country (52).

The biological relevance of a potential correlate of protection such as the MGIA can only be confirmed by demonstrating an association with *in vivo* protection from either a controlled human mycobacterial infection or the natural development of TB disease. Deliberate human infection models, while commonly used for other infectious diseases such as malaria, are not ethically viable for virulent *M.tb*. We have thus previously developed and described an *in vivo* human infection model using intradermal BCG vaccination as a surrogate challenge agent (53–55). Having established a standardised and transferable protocol for the direct PBMC MGIA, we took steps in 2020 to biologically validate the assay against *in vivo* controlled human infection studies with attenuated *M. bovis* as a surrogate for virulent *M. tb* (56). It was confirmed in two independent cohorts that control of mycobacterial growth in the direct PBMC MGIA is enhanced in BCG-vaccinated compared with naïve volunteers. Importantly, in both cohorts, this control correlated with protection from *in vivo* human infection at a group level and at the level of individual volunteers within the BCG-vaccinated groups. This was the first report of any MGIA correlating with *in vivo* protection in the species of interest, humans, and furthermore at this level of granularity (56).

In addition to measuring peripheral responses, a lung cell based MGIA would be valuable given the growing body of evidence suggesting that early localised immune responses within the lung may be most relevant in mediating protection from TB (57). Furthermore, vaccine delivery by the mucosal route may enhance immunogenicity and protective immunity (57–59), and lung responses also appear to be important for vaccines given by routes other than aerosol (60). In 2018, Radloff et al. attempted to adapt the direct MGIA for human bronchoalveolar lavage cells (BALC) using 1x10^6^ cells and an inoculum of 58,000 CFU of *M.tb* co-cultured for 96 hours (61). They did not observe an improvement in control of *M.tb* growth by either fresh PBMC or BALC following BCG vaccination, although the mycobacterial input was significantly higher than other direct MGIAs reported (58,000 CFU vs. the typical range of 100-500 CFU), which has been previously shown to overwhelm the host immune response and may be a particular issue when using a more virulent mycobacterial strain as described here. The authors suggest that their ability to detect an effect of Vitamin D3 supplementation on mycobacterial growth confirms assay functionality, but this should be interpreted with caution given the likely differences in sensitivity required to observe an effect of a mediator added directly to the co-culture compared with a complex and dynamic immune response induced *in vivo* (61).

Recent attempts to further optimise the human direct MGIA have included a comparison of quantifying *M.tb* growth at the end of the co-culture period using TTP by BACTEC MGIT compared with traditional CFU on agar and relative fluorescence signal (RFU) obtained by live-cell microscopy (62). All three quantification methods correlated strongly with one another, offering a live-cell imaging-based direct MGIA as a promising alternative during which cell viability can be continuously monitored (62).

##### 3.2.1.2 Murine direct MGIA

Novel TB vaccine candidates are currently evaluated using preclinical models, most frequently mice, prior to progression into larger animal models and ultimately clinical trials. Such testing requires that animals are ‘challenged’ (infected) with virulent *M.tb* to assess vaccine-induced protection. The murine MGIA provides an alternative measure of vaccine efficacy in line with the 3Rs principles of Replacing, Refining and Reducing the use of animals in research (4). It also offers an opportunity to biologically validate the assay by correlating outcomes with *in vivo* protection.

We reported the first application of the direct MGIA to the murine model in 2013 (63). Using a protocol of 1x10^6^ splenocytes co-cultured with ~500 CFU BCG Pasteur in sealed 2ml rotating tubes for 96 hours, we observed enhanced control of BCG growth by splenocytes from BCG-vaccinated compared with naïve mice (n=8 mice per group) consistent with *in vivo* protection (63). Further optimisation demonstrated an effect on assay outcome of BCG strain differences, mycobacterial inoculum, and number of splenocytes. It was determined that 5x10^6^ splenocytes and ~100 CFU BCG resulted in a comparatively large and statistically significant difference between groups while retaining acceptable levels of intra-assay variability (64). Co-culturing with the fast-growing *M. smegmatis* also led to enhanced growth inhibition following BCG vaccination, and could represent a more rapid direct MGIA method (64).

In 2016, Yang et al. described the direct MGIA with modifications to the multiplicity of infection (MOI) (5x10^6^ splenocytes infected with ~500 CFU BCG) and co-cultures performed in 48-well tissue culture plates. Furthermore, 5 replicate co-cultures were prepared by pooling splenocytes recovered from 3 mice for each of the BCG vaccinated and naïve groups (65). Using these methods, splenocytes from BCG vaccinated mice showed significantly enhanced control of mycobacterial growth whether quantified using the BACTEC MGIT system or CFU plating. This inhibition was observed at 1 week and 5 weeks post-BCG vaccination but not at 29 weeks (65). The following year, Jensen et al. applied the direct MGIA using 50 CFU of *M.tb* Erdman co-cultured with 5x10^6^ splenocytes in sealed 2ml tubes (66). Optimisation efforts demonstrated that cell viability at 96 hours could be improved by enriching the culture media with nutrients, and by not rotating the cultures. Under these conditions, mycobacterial growth was significantly inhibited using splenocytes from BCG vaccinated vs. naïve mice, and was shown to be reproducible both within and between experiments (66).

Given the potential relevance of local lung immunity discussed in Section 3.1.1, we recently reported an adaptation of the direct MGIA for use with murine lung cells (67). Using a lung cell input of 1x10^6^ co-cultured with ~100 CFU BCG in 48-well tissue culture plates and 4 technical replicates from each group of 6 pooled mice, control of mycobacterial growth was significantly improved in mice following subcutaneous (SC) BCG, intranasal (IN) BCG, or BCG SC with a mucosal boost, compared with naïve mice (67). To permit a more direct comparison with *in vivo* outcomes, the assay was also optimised for an *M.tb* Erdman inoculum. The addition of the BCG growth inhibitor 2-thiophenecarboxylic acid hydrazide (TCH) circumvented the confounding quantification of residual BCG from IN immunisation and increased sensitivity to observe a reduction in *M.tb* CFU in the MGIA of the IN group (67).

##### 3.2.1.3 Non-human primate direct MGIA

NHPs are widely considered the most representative model for human TB due to their physiological similarities and the clinical similarities in the course of *M.tb* infection (68), with a recent emphasis on the use of NHPs as a ‘gatekeeper’ for progression of TB vaccine candidates to clinical trials (69). Due to this and other factors, the number of animals used in the field is increasing. The use of NHPs in medical research is emotive, and an MGIA would provide a high-throughput refinement to early testing of TB vaccine candidates as an alternative to *in vivo* infection with pathogenic *M.tb*.

In 2021, we reported an adaptation of the direct MGIA using whole blood, in which enhanced mycobacterial control was observed following BCG vaccination in cynomolgus macaques, consistent with *in vivo* data demonstrating a partially-protective effect of BCG in this species (70). The kinetic of response with a peak at 8 weeks post-vaccination that waned by 24 weeks was consistent with that previously observed in the human direct MGIA (43). Steps were taken to optimise the assay for use with cryopreserved PBMC, with intra-assay repeatability and sensitivity to detect BCG vaccine-induced control improved by increasing cell concentration or mycobacterial input, and by co-culturing in 48-well plates rather than a closed tube system, in line with findings using human and mouse cells (50, 64, 70). Standardisation and harmonisation efforts resulted in high consistency agreements, with repeatability and intermediate precision <10% CV and inter-site reproducibility <20% CV, although some systematic differences were observed (70). Further description of optimisation experiments and a detailed protocol for this assay has been published (51).

The NHP MGIA offers a unique opportunity for biological assay validation at the individual animal as well as group level ie. samples can be run in the MGIA prior to *in vivo M.tb* challenge, and data on protection from *in vivo* challenge then obtained from the same animals. This is not possible in mice due to euthanasia required for splenocyte harvest for MGIA, or in humans as they cannot ethically be challenged with *M.tb* (although attenuated *M. bovis* BCG may be used as a surrogate as described in Section 3.1.1). We demonstrated a correlation between MGIA outcome and measures of protection from *in vivo* disease development following challenge with either ID attenuated *M. bovis* BCG or aerosol/endobronchial *M.tb* at a group and individual animal level in three different NHP cohorts (70).

In an additional study in cynomolgus macaques of Chinese origin, control of mycobacterial growth was observed in 3 of the 6 BCG vaccinated animals following vaccination but none of the unvaccinated animals (71). Interestingly there were no significant associations between MGIA outcome and measures of protection, although there was a trend towards an association between mycobacterial growth at 8 weeks post-vaccination and X-ray score (71). A key difference between this and the previously-described study where outcomes correlated with protection is that the latter reported ‘vaccine response’ (post-vaccination mycobacterial growth minus baseline mycobacterial growth) rather than raw post-vaccination growth which may be confounded by pre-existing baseline immunity (70). Further work on the NHP direct MGIA is required to establish the most meaningful read-out, improve assay sensitivity, evaluate performance in trials of novel TB vaccine candidates and interrogate immune mechanisms of control.

##### 3.2.1.4 Cattle direct MGIA

Bovine TB (bTB), caused by *Mycobacterium bovis* (*M. bovis*), is a major problem affecting livestock with considerable economic impact worldwide. *M. bovis* can also infect and cause zoonotic disease in humans, mainly through the consumption of unpasteurised milk (72). BCG vaccination confers a degree of variable protection in cattle that is comparable to that seen in humans (73). However, use of BCG is not currently permitted for the control of bTB in the UK due to a high degree of antigen homology with *M. bovis*, leading to an inability to distinguish between a vaccinated and infected animal using the Tuberculin Purified Protein Derivative (PPD) skin test (72). A new, more effective bTB vaccine is urgently needed. Furthermore, cattle represent a model organism for studying *M.tb* including for testing novel TB vaccine candidates (74). Hampered by the same issues of a lack of immune correlate of protection from bTB in cattle and feasibility and ethical limitations on animal numbers and severity of *in vivo* challenge experiments, an MGIA for use in this species would be of considerable benefit to the field.

However, attempts to adapt the whole blood and direct PBMC MGIAs for use in cattle have to date been unsuccessful. Pepponi et al. were unable to detect a difference in mycobacterial growth between BCG vaccinated and naïve Holstein-Friesian cattle using either the whole blood or the direct PBMC MGIA at any of the time-points evaluated, although there was a trend towards improved growth inhibition in the vaccinated group at 9 and 12 weeks post-BCG using the PBMC assay (75). The whole blood assay suffered from timepoint-dependent batch effects in both groups which may be due to the young age of the calves at the onset of the study, or to technical variability from real-time processing as previously seen with the human direct whole blood MGIA (43).

It is unclear whether the lack of vaccine effect observed in the cattle direct MGIA represents a technical issue or is a true reflection of the biological situation – indeed, levels of *in vivo* protection in these animals is unknown, and unlike laboratory species, they are more likely to have faced challenge by environmental mycobacteria or *mycobacterium avium* subsp. *paratuberculosis* (MAP) infection and are less genetically homogenous (75). Although the direct MGIA may not be suitable for transfer across all species, particularly those that are immunologically distinct, further work is required before its potential in cattle is ruled out. The authors did perform some assay optimisation, demonstrating no effect of blood storage temperature and an improvement in intra-assay consistency between replicates by eliminating the centrifugation step following cell lysis (75), which is also of relevance to the assay in the context of other species.

#### 3.2.2 Non-specific effects of BCG vaccination

##### 3.2.2.1 Control of heterologous bacteria

The potential non-specific effects of BCG vaccination, including a reduction in all-cause mortality in infants, have been discussed elsewhere (76–78). In 2022, we published the findings of a randomised controlled clinical study evaluating the functional non-specific effects of BCG vaccination in UK adults (79). Using a similar protocol as described above (3x10^6^ PBMC in 600µl co-culture medium in 48-well tissue culture plates followed by water lysis), the direct MGIA was adapted to evaluate control of each of four common bacteria, and responses compared at baseline and 2, 7, 14, 28 and 84 days post-BCG vaccination. In addition to a significant specific MGIA effect following BCG vaccination, we observed significantly improved control of the Gram-negative bacteria *Escherichia coli* and *Klebsiella pneumonia*, but not the Gram-positive bacteria *Staphylococcus aureus* and *Streptococcus agalactiae*. There was also a modest association between *S. aureus* nasal carriage and growth of *S. aureus* in the GIA (79). Our findings suggest that such direct GIAs represent a tractable model for the investigation of the immune mechanisms underlying the non-specific as well as specific effects of BCG vaccination. Further to this, Joosten et al. demonstrated that the effects of trained innate immunity can be detected using the direct PBMC MGIA and may contribute to the control of mycobacterial growth observed early after exposure to *M. tb* (47).

##### 3.2.2.2 Bladder cancer

Another application of BCG that results from off-target immunomodulatory effects is as a cancer therapy, and its efficacy against bladder cancer has now been confirmed (80). Bilsen et al. performed the direct MGIA using PBMC from two patients with high-risk, non-muscle-invasive bladder cancer that had developed systemic BCG disease following BCG instillation (81). One patient, who had high-grade fever without organ involvement, showed a moderate mycobacterial antigen-specific T-cell response, but enhanced mycobacterial control in the MGIA compared to healthy controls. The other patient, who presented with sepsis and organ involvement, had a higher antigen-specific T-cell response but reduced mycobacterial control in the MGIA. Although interpretation is limited by the very small sample, the authors suggest that patients with poor control of mycobacterial growth in the MGIA could benefit more from tuberculostatic drugs (81). A more comprehensive application of the direct MGIA in the context of BCG therapy for bladder cancer would be of interest.

#### 3.2.3 Evaluating TB vaccine candidates

As demonstrated, observing a BCG vaccine effect in the direct MGIA for species where BCG is known to confer protection *in vivo* is a useful benchmark on which to optimise assay conditions. It also offers a crucial positive control for confirming assay performance when responses in experimental groups are unknown. However, ultimate utility of the assay as a tool for candidate TB vaccine evaluation lies in its ability to detect biologically meaningful functional responses to other TB vaccines. Several groups have explored this in the context of both prophylactic and therapeutic vaccine candidates.

##### 3.2.3.1 Prophylactic TB vaccine candidates

In the study described in section 3.1.2, Yang et al. also measured the protective activity of 3 different vaccine preparations in mice using both the direct splenocyte MGIA and *in vivo* challenge with virulent *M.tb* (65). Mice were vaccinated with either BCG, BCG formulated in a DDA/TDB adjuvant, or the ESAT6-Antigen 85B (SD1) fusion protein suspended in DDA/TDB adjuvant. At 6 weeks post-vaccination, mice were either challenged with *M.tb* by the aerosol route (followed by quantification of mycobacterial burden 4 weeks later), or splenocytes were harvested for the direct MGIA (65). Both methods detected significant improvement in mycobacterial control in all vaccinated groups compared with unvaccinated controls, and MGIA outcome mirrored the pattern of differences between groups observed following *in vivo* challenge with a highly significant correlation. The authors concluded that this validation supports the use of the murine direct MGIA for screening novel TB vaccine candidates and investigating immune protective mechanisms (65). It should be noted that splenocytes from 3 mice per group were pooled and distributed among 5 replicate MGIA co-cultures in this study, which improved reproducibility but may be considered a limitation as biological variability is unaccounted for.

Having optimised the direct splenocyte MGIA for use with a virulent *M.tb* inoculum, Jensen et al. tested experimental vaccine regimens developed at Statens Serum Institut (SSI) which had previously shown protective efficacy in *in vivo* challenge experiments (66). Mice were immunised with either BCG alone, H56:CAF01 (previously shown to confer ~1 log_10_ CFU protection *in vivo*), or H56:CAF01 SBS with BCG (previously shown to confer ~1.3 log_10_ CFU protection *in vivo*). A significant improvement in the control of mycobacterial growth in the MGIA was observed in all groups, with H56:CAF01 alongside BCG inducing the strongest inhibition compared with the placebo group, corresponding to relative *in vivo* protection (66). Interestingly, the adjuvant-alone control group mediated significant growth inhibition at the level of BCG, which Joosten et al. suggest may fit with their observations of induced innate trained immunity (see Section 5.3.1) (47).

Using the direct splenocyte MGIA and the adaptation for use with murine lung cells, Painter et al. compared growth inhibition of *M.tb* Erdman conferred by vaccination with either SC or IN BCG, or with IN BCG boosted with the candidate vaccine spore-FP1 three weeks later. MGIA outcomes in both compartments were consistent at the group level with existing literature on levels of *in vivo* protection conferred by these regimens (67). In 2022, Pujilestari et al. applied the murine direct splenocyte MGIA, and the first description of the direct MGIA using murine PBMC, to assess the efficacy of the TB vaccine candidate pcDNA3.1-rpfB. Control of mycobacterial growth by splenocytes was not different in mice vaccinated with pcDNA3.1-rpfB compared with a control group vaccinated with pcDNA3.1, or from BCG vaccinated mice, while in PBMC, pcDNA3.1-rpfB conferred superior control of mycobacterial growth compared with pcDNA3 but was not different from BCG (82). However, interpretation of this study is limited by the lack of an unvaccinated group to confirm a BCG vaccine effect as a positive assay control. A follow-up study tested a similar construct using resuscitation-promoting factor D (*rpfD*) rather than *rpfB*, and this time an additional control of *M. tb* incubated with media alone and no splenocytes was included (83). Control of mycobacterial growth was enhanced in pcDNA3.1-rpfD-vaccinated mice compared with pcDNA3.1-alone in both splenocyte and PBMC MGIAs, but again this was not significantly different from the BCG group (83).

In 2013, we reported the evaluation of a human BCG challenge model to assess antimycobacterial immunity induced by BCG and the candidate TB vaccine, MVA85A, in which the direct MGIA was performed on the day of challenge (53). The whole blood MGIA detected no significant differences between volunteers that were BCG and MVA85A naïve, had received prior BCG but no MVA85A vaccination, prior BCG followed by MVA85A vaccination, or MVA85A with no prior BCG vaccination (53). Conversely, the direct MGIA using cryopreserved PBMC detected significantly improved control of mycobacterial growth in the historically BCG-vaccinated group compared with the naïve or the MVA85A alone groups (56), which is consistent with the *in vivo* result where volunteers with a history of BCG showed some degree of protective immunity to intradermal BCG challenge (53). The disparity between outcomes using whole blood and PBMC may be due to greater inter-assay variability in the whole blood assay (43), the confounding influence of haemoglobin and iron (84), or the further assay optimisation that was performed in the interim period between studies (50).

More recently, the whole blood direct MGIA has been applied in two Phase 1 trials of novel TB vaccine candidates. Hoft et al. reported on the safety and immunogenicity of the recombinant BCG vaccine AERAS-422 (which over-expresses the *M.tb* antigens Ag85A, Ag85B and Rv3407 and expresses mutant perfringolysin) in a double-blind trial in healthy BCG-naïve adults (85). Volunteers were randomised to receive a single dose of either AERAS-422 or non-recombinant TICE BCG. Significantly enhanced mycobacterial growth inhibition was observed at days 84 and 182 following vaccination in the high dose AERAS-422 group, but not the Tice BCG vaccinated or low dose AERAS-422 group (85). Sagawa et al. reported on the safety, tolerability and immunogenicity of a thermostable lyophilised single-vial presentation of the ID93+GLA-SE vaccine candidate compared with the non-thermostable two-vial vaccine presentation in healthy adults (86). *M.tb* growth in whole blood was assessed at days 0, 70 and 224 but neither vaccine presentation induced a significant enhancement in growth inhibition compared with paired pre-vaccinated responses (86).

##### 3.2.3.2 Therapeutic TB vaccine candidates

In recent years, Prabowo et al. have applied the direct MGIA to evaluate therapeutic TB vaccine candidates and explore underlying immune mechanisms of mycobacterial control (49, 87). In an initial study in which human PBMC were co-cultured with ~100 CFU BCG using the in-tube direct MGIA, a history of BCG vaccination was associated with enhanced ability of isoniazid (INH) and rifampicin (RIF) to control mycobacterial growth at certain concentrations (49). The finding for INH (but not RIF) was replicated using the murine direct splenocyte MGIA (49). MGIA responses were also measured in mice following one or two vaccinations with RUTI, one of the leading therapeutic TB vaccines in the clinical pipeline (87). Using the conditions of 5x10^6^ splenocytes co-cultured with 90 CFU BCG in 48-well tissue culture plates, a significant improvement in the control of mycobacterial growth was observed one week after the first vaccination and 3 weeks after the second vaccination with RUTI compared with baseline. In a separate experiment using the in-tube protocol, RUTI-induced control of mycobacterial growth was superior to BCG-induced control at 6 weeks post-vaccination (87).

### 3.3 Application of the direct MGIA to the study of clinical cohorts

One advantage of an unbiased ‘sum-of-the-parts’ assay using primary cells or blood samples is its ability to represent the immune condition present in a given individual, or group of individuals, at the time of sampling. As discussed, this can be utilised for assessing the effects of drugs and vaccines, but it may also be exploited to study ability to control mycobacterial growth across different clinical cohorts, including the effects of *M.tb* exposure/infection, TB disease, or other comorbidities. Findings may inform mechanistic understanding as well as the prioritisation of groups for interventions such as vaccination or drug therapy.

#### 3.3.1 Patients with latent TB infection or active TB disease

In the early Cheon et al. study, the direct whole blood MGIA was performed using blood from a group of healthy tuberculin skin test (TST)-positive (n=4) and TST-negative (n=4) US adults. Samples were co-cultured with three strains of *M.tb* (H37Ra, H37Rv and MP-28) with growth assessed at 24 hour intervals up to 96 hours. The strains differed in their ability to grow in the blood consistent with their relative virulence, with a trend towards improved inhibition of H37Ra in TST-positive compared with TST-negative individuals (42).

In 2018, we employed the direct whole blood MGIA to generate a more comprehensive functional profile of growth control among UK adults with confirmed latent TB infection (LTBI), active TB disease, or uninfected controls (88). Using either BCG Pasteur or *M.tb* H37Rv as the inoculum, the assay was able to discriminate between individuals with different states of *M.tb* infection; control of mycobacterial growth was greatest in those with active TB disease and poorest in healthy controls. We hypothesised that superior control may be associated with immune activation resulting from high *in vivo* bacillary loads in the TB patients (88). The LTBI group displayed an intermediate ability to control with a wide spectrum of MGIA responses that overlapped with both the active disease and uninfected control groups, suggestive of some individuals with a high bacillary burden but subclinical disease demonstrating enhanced immune activation, and those with quiescent infection clustering with the healthy controls (88).

This was mirrored in a study of macaques, in which control of mycobacterial growth in the NHP direct PBMC MGIA was significantly improved at 6 and 12 weeks post experimental *M.tb* infection, although divergent TB disease outcomes in rhesus and cynomolgus species were not reflected in the MGIA (89). While control of BCG growth in the direct PBMC MGIA did not differ between LTBI individuals, TB patients or uninfected controls in the aforementioned study of Dutch volunteers, enhanced ability to control mycobacterial growth was observed in a subgroup of individuals with recent exposure to *M.tb* (47). This further supports the idea that recent mycobacterial sensitisation drives a more effective immune response, and that this is reflected in the direct MGIA.

However, the situation may differ in settings with high or intermediate TB burdens where ongoing high levels of mycobacterial sensitisation could impart broad inhibition of mycobacterial growth irrespective of *M.tb* status, thus masking any potentially augmentative effects of mycobacterial sensitisation such as LTBI or BCG vaccination (90). Indeed, there was no difference in direct whole blood MGIA outcome between individuals with LTBI and uninfected controls in a cross-sectional study of South African adults, young adults and children; or between TST negative and positive children in India (90, 91). This hypothesis is supported by the lack of difference in innate and specific T cell responses between *M.tb* infected and uninfected individuals in the South African study (90). It is possible that background sensitisation also influenced outcomes in a study of individuals in the intermediate burden setting of South Korea, which reported superior control of BCG growth in uninfected controls compared with LTBI individuals using the direct PBMC MGIA (45). Such contrasting findings may also reflect differences in assay conditions, highlighting the need for a standardised consensus protocol.

#### 3.3.2 Comorbidities

##### 3.3.2.1 Helminth infection

There is considerable geographical overlap between soil-transmitted helminths and *M.tb*, and it is estimated that between 20 and 35% of TB patients are co-infected with helminths in TB endemic regions (92). While *M.tb* infection induces a proinflammatory Th1 and Th17 response, helminth infection drives a Th2 anti-inflammatory response (93, 94). It has been suggested that in co-infected individuals, helminth-induced immunomodulation promotes progression to active TB disease, exacerbates TB pathology and reduces BCG vaccine efficacy (95–97). Perhaps unexpectedly, we found that Nepalese migrants to the UK with hookworm infection had enhanced ability to control *M.tb* growth in the direct whole blood MGIA which was lost following hookworm treatment (98). There was a significant inverse correlation between mycobacterial growth and eosinophil count, and transcriptomic analysis revealed that eosinophils were the primary contributor to the gene expression signature. The authors propose a potential anti-mycobacterial role for helminth-induced eosinophils which may account for the reduced prevalence of LTBI among hookworm-infected individuals (98).

##### 3.3.2.2 Diabetes

More recently, Bobadilla-del-Valle et al. applied the direct whole blood MGIA to evaluate the immune response in patients with type-2 diabetes mellitus (DM2) with optimal and poor glycaemic control (99). They found significantly reduced control of *M.tb* H37Rv growth among DM2 patients compared with healthy volunteers, and among DM2 patients with poor glycaemic control (but not those with optimal glycaemic control) compared with healthy volunteers (99). This is consistent with the well-documented increased risk of TB in DM2 patients, particularly those with poor glycaemic control. The authors point to the potential utility of the direct whole blood MGIA as an *in vitro* marker of *M.tb* control *in vivo* in DM2 patients, and as a tool for evaluating individual mycobacteria-specific immune responses to inform host-directed therapy selection (99). Kewcharoenwong et al. employed a modified direct MGIA to assess the bactericidal capabilities of primary human monocytes from patients treated with and without the anti-diabetic drug glibenclamide (a blocker of ATP-sensitive potassium channels) (100). In this assay, cells from DM2 patients who were being treated with glibenclamide showed reduced ability to inhibit BCG and *M.tb* growth compared with those being treated with other anti-diabetic drugs, or cells from healthy controls. This impairment of antimycobacterial function of monocytes was associated with reduced M1 and enhanced M2 polarisation, consistent with previous links between M2 macrophages and reduced microbicidal activity and heightened susceptibility to TB (100).

### 3.4 Employing the direct MGIA to identify potential immune correlates of protection

In addition to its applications in drug/vaccine evaluation and assessment of clinical cohorts, the direct MGIA is proving a useful tool for delineating the immune mechanisms that contribute to the control of mycobacterial growth. The simplicity of the assay makes it a tractable model for teasing apart relative contributions of different immune parameters, through for example manipulating cell ratios or serum factors (52, 101). Findings may contribute to the identification of immune correlates of protection from TB, although *in vivo* validation following vaccination with a highly efficacious regimen would clearly be required.

#### 3.4.1 Cellular immune mechanisms

##### 3.4.1.1 T cells and proinflammatory cytokines

The initial Cheon et al. study (see Section 3.1.1) described the effect of T cell depletion on control of *M.tb* H37Ra in the direct whole blood MGIA using 24 and 96 hour co-cultures in PPD+ and PPD- donors. There was no effect of depleting either CD4+ or CD8+ T cells alone for any of the conditions, apart from for CD8+ T cells in PPD+ donors at 24 hours and in PPD- donors at 96 hours (42). When both CD4+ and CD8+ T cells were depleted together, there was a significant effect after both 24 and 96 hour co-cultures for PPD+ but not PPD- individuals. However, no such effects were seen for the clinical *M.tb* isolate MP-28. Prabowo et al. found no significant correlations between the frequencies of BCG-specific CD4+ and CD8+ T cells and mycobacterial growth in the direct PBMC MGIA (48).

In recently-exposed individuals, CD4+ central memory T cells correlated strongly with ability to control mycobacterial growth, although there was an inverse association between mycobacterial control and the percentage of CD3+ T cells, as well as CD4+ and CD8+ effector cells (47). Interestingly, activated T cells have been shown to associate with reduced control of mycobacterial growth in the direct PBMC MGIA (48), consistent with recent evidence that activated T cells represent a correlate of risk of TB disease.

The potential association between mycobacterial growth control and IFN-γ responses has been explored in several direct MGIA studies with mixed results. Prabowo et al. described a significant inverse correlation between IFN-γ ELISpot response and lower mycobacterial growth (48). Jensen et al. also found a strong significant inverse correlation between IFN-γ release into the co-cultures and mycobacterial growth inhibition in the murine splenocyte direct MGIA based on the mean values for groups receiving different vaccination regimens (66), and Zelmer et al. demonstrated reduced ability to control mycobacterial growth in IFN-γ KO mice (64). Interestingly, le Roex et al. applied the direct MGIA to whole blood from African buffalo and found that animals with a strong IFN-γ response following bovine and avian PPD stimulation were better able to restrict mycobacterial growth (102).

Conversely, we did not see an association between IFN-γ ELISpot and MGIA outcome at most time-points in our study of primary BCG vaccination/revaccination (43), or the EURIPRED study across three laboratories (50). The Joosten et al. and Baguma et al. studies in Dutch and South African populations respectively also found no association between MGIA activity and CD4+ or CD8+ T cells producing IFN-γ (47, 90). Prabowo et al. suggest that IFN-γ producing NK cells may have contributed to the correlation observed between ELISpot and MGIA, particularly given the association between mycobacterial growth inhibition and NK cell frequency in this study (see Section 3.4.3.2) (48), and this may account for the lack of association with IFN-γ producing T cells alone.

The effect of other cytokines has also been explored, albeit to a lesser extent. Cheon et al. described the addition of methylprednisone or pentoxifylline to direct whole blood MGIA co-cultures to reduce TNF-α expression, which was associated with increased growth of *M.tb* H37Ra (but not MP-28) in PPD+ but not PPD- reactors (42). Prabowo et al. found a significant inverse correlation between IL-10 production (but no association with IFN-γ, IP-10, TNF-α, IL-12, GM-CSF, IL-6 or IL-17) and control of mycobacterial growth (48), although Jensen et al. did not see associations with IL-10 (or IL-6) in the murine splenocyte direct MGIA (66). Also in mice, Marsay et al. described correlations between control of mycobacterial growth and levels of the proinflammatory genes IFN-γ, IL-15Ra, CXCL9, CD70 and Ikbkg, while genes in the lysosome pathway were associated with lack of control (63). O’Shea et al. noted significant associations between control of *M.tb* growth and serum concentrations of Gro, TGF-α, PDGF-BB, PDGF-AA, IP-10 and MDC in LTBI individuals, TB patients and healthy controls (88).

##### 3.4.1.2 Polyfunctional T cells

Smith et al. noted that BCG vaccination induced predominantly polyfunctional IFN-γ+ TNF-α+ IL-2+ CD4+ T cells in a UK infant cohort, and that the frequency of these cells (but not IL-17+ CD4+ T cells) correlated with control of mycobacterial growth in the direct PBMC MGIA (46). Consistent with these findings, we observed IFN-γ+ TNF-α+ IL-2+ CD4+ T cells to be the dominant responder population at 2 weeks post-BCG infection in a human mycobacterial challenge model, and this cell subset correlated with control of mycobacterial growth in the direct MGIA when all groups were combined with a non-significant trend towards a correlation in the BCG vaccinated group alone (56). There were also significant correlations between control of mycobacterial growth and IFN-γ, TNF-α, IL-2 and IL-17 quadruple-cytokine producing CD4+ T cells, and all permutations of triple-cytokine producing cells. The strongest association in the BCG vaccinated group was with IFN-γ and TNF-α double-cytokine producing cells, which also represented one of the largest cell populations (56).

However, this finding was not replicated in the cohort of individuals from different settings in the Netherlands (47) or in healthy controls and LTBI participants from Korea (45), although it should be cautioned that frequencies of polyfunctional T cells in both studies were very low, and conclusions may be confounded by combining different groups in the correlation analysis. While Jensen et al. reported a significant association between control of mycobacterial growth in the murine direct splenocyte MGIA and polyfunctional CD4+ T cells in H56:CAF01-vaccinated mice, they suggested the association may be driven by one outlier, and the slope was null following exclusion of this data point (66). It is possible that such a discrepancy is due to the measurement of effector responses soon after vaccination/exposure in these studies, while our controlled human infection model (CHIM) study also captured vaccine-induced memory responses re-stimulated by *in vivo* infection (56).

#### 3.4.2 Humoral immune mechanisms

Due to the intracellular nature of mycobacteria and the demonstration of specific T cells as the pillar of acquired immunity to TB, the humoral immune response has been largely understudied in TB vaccine research and development (R&D) (103). However, recent evidence suggests that B cells and antibodies may play a more significant role than previously appreciated (104), and their potential contribution in the context of the MGIA is starting to be explored. Joosten et al. noted that capacity to control BCG in the direct PBMC MGIA in recently exposed individuals correlated with the percentage of CD19+ B cells (47). Furthermore, we described significant elevation of activated and atypical memory B cells among individuals with LTBI and active TB compared with healthy controls, and an association between frequency of these cells (but not total B cells) with control of mycobacterial growth in the human direct whole blood MGIA (88).

Using samples from the UK BCG vaccination and revaccination cohort previously described (43), IgG responses correlated significantly with mycobacterial growth inhibition at 4 weeks post-vaccination (105). In the human mycobacterial challenge study, we identified a significant correlation between levels of plasma IgG (but not IgA or IgM) at 2 weeks post-BCG infection and control of mycobacterial growth at day of challenge (56). IgG1 (but not IgG2) responses to *M.tb*-specific antigens also correlated with improved MGIA control in our study of healthy, LTBI and active TB patients (88). More recently, Bitencourt et al. demonstrated a functional contribution of BCG-induced antibodies in the direct MGIA (52). When serum was matched to time-point there was a significant improvement in control of mycobacterial growth at 84 days post-BCG vaccination compared with baseline. When serum was swapped (baseline PBMC cultured with day 84 serum and day 84 PBMC with baseline serum), control of mycobacterial growth no longer differed between time-points. This finding was validated in an independent cohort (52).

#### 3.4.3 Innate immune mechanisms

##### 3.4.3.1 Monocytes and M:L ratio

Monocytes serve as the primary niche for *M.tb* infection and also participate in the inflammatory response and control of mycobacterial growth (106). Cross et al. reported that the rate of *M.tb* growth was increased in monocyte-depleted and CD66b+ neutrophil-depleted compared to un-depleted whole blood, although the inverse was true in the presence of rifampicin (107). In the O’Shea et al. study, mycobacterial growth inhibition was associated with more intermediate and fewer non-classical monocytes and altered subset frequencies that was normalised following successful anti-TB treatment (88). However, in their cohort of naïve and BCG vaccinated UK volunteers, Prabowo et al. found no associations between control of mycobacterial growth and frequencies of monocytes, M1 monocytes, M2 monocytes, M1/M2 ratio, CD64+ monocytes, CD123+ monocytes or suppressor monocytes (48). Baguma et al. also reported no association between control of *M.tb* growth in the direct whole blood MGIA and numbers of monocytes, neutrophils or myeloid dendritic cells, nor uptake of GFP-expressing BCG by these cells in the high TB burden population (90).

Joosten et al. performed a more comprehensive analysis in cells from their combined mixed cohort of healthy, BCG vaccinated, LTBI and active TB patients, reporting an association between mycobacterial control in the direct PBMC MGIA and the presence and activity of a non-classical CD14^dim^ monocyte subset, particularly those producing the chemokine CXCL10 (47). Addition of a CXCR3 receptor agonist to the co-cultures including PBMC that otherwise controlled mycobacterial growth abrogated the inhibition, suggesting functional involvement of the CXCR3/CXCL10 axis. Monocytes were unable to control mycobacterial growth in the absence of T cells, indicating the requirement for T cells in this process. Finally, cytokines associated with trained innate immunity were found at increased levels in MGIA supernatants of recently *M.tb*-exposed individuals (47).

The ratio of monocytes to lymphocytes (M:L ratio) has previously been associated with risk of TB disease in a series of prospective cohort studies (108–110). Consistent with this, Naranbhai et al. showed that an increased M:L ratio *in vivo* correlated with reduced ability to control mycobacterial growth in the direct whole blood MGIA using blood from healthy adult donors (101). The association was further explored by mixing monocytes and lymphocytes in the direct PBMC MGIA at ratios approximating the 25^th^, 50^th^ and 75^th^ centiles of *in vivo* M:L ratios. Interestingly, while a higher *in vitro* M:L ratio was associated with reduced ability to control mycobacterial growth, individuals with a higher M:L ratio *in vivo* had poor control regardless of the M:L ratio created in the MGIA. This suggests a qualitative effect of monocytes from individuals with higher M:L ratios *in vivo* being relatively inferior at inhibiting mycobacterial growth which dominates over quantitative differences in explaining MGIA outcome (101).

In the O’Shea et al. study of LTBI and active TB patients, as well as the Joosten et al. study of BCG vaccinated, recently-exposed, LTBI and TB patients, M:L ratio was negatively associated with mycobacterial growth (47, 88). Interestingly, O’Shea et al. found that this converted to a positive association following TB treatment, consistent with the Naranbhai et al. findings in healthy individuals (101). Prabowo et al. reported no association between M:L ratio and MGIA outcome (48), although it should be noted that the ratio was defined differently among the different studies.

In an adaptation of the murine direct MGIA, bone marrow-derived macrophages (BMDM) were co-cultured with BCG for 96 hours in a closed tube system (111). Macrophages from *Nos2^-/-^ mice* (which lack iNOS protein) showed a significantly reduced ability to control mycobacterial growth compared with those from wild-type mice. In contrast, macrophages from *Gch1fl/fl*Tie2cre mice (which lack *Gch1* expression, preventing the biosynthesis of BH4 – a required cofactor for iNOS NO production) mediated significantly improved mycobacterial control (111). Using samples from BCG-vaccinated infants, this finding was further supported by a significant inverse correlation between *Gch1* expression and control of mycobacterial growth in the direct PBMC MGIA, indicating NO-independent functions of *Gch1* in mycobacterial control (111).

##### 3.4.3.2 Natural killer cells

Prabowo et al. described a significant correlation between the frequencies of NK cells and production of perforin and enhanced control of mycobacterial growth in both naïve in BCG-vaccinated individuals (48). In the aforementioned study of Cross et al., the rate of *M.tb* growth was increased in NK-depleted compared to un-depleted whole blood in the absence of rifampicin (107). Using the cattle direct PBMC MGIA, Pepponi et al. found no association between the frequency of NK cells (or CD14+ monocytes) and control of mycobacterial growth, although there was a significant association for NK T-like cells (CD3+CD335+) (75). This is consistent with evidence in humans and mice that NKT cells may contribute to protection from TB (112). In the Phase 1 trial of AERAS-422, the development of robust vaccine-induced mycobacterial growth inhibition at 84 days post-vaccination was associated with robust induction of NK and cytotoxicity gene expression modules in PBMCs at 14 days post-vaccination (85).

##### 3.4.3.3 Granulocytes

In the hookworm study of O’Shea et al., control of mycobacterial growth in the direct whole blood MGIA correlated with eosinophil count (98). Given that eosinophil frequencies were elevated in this hookworm-infected cohort, and others have shown that these cells are activated by mycobacteria and accumulate at sites of mycobacterial infection, the authors suggest that hookworm-induced eosinophilia may have a role in defence against *M.tb* (98). Indeed, eosinophils are known to have anti-mycobacterial activity through the secretion of cytotoxic proteins (113, 114). However, the direct PBMC MGIA also showed enhanced control of *M.tb* growth in hookworm infected individuals despite lacking polymorphonuclear cells, indicating the contribution of other immune mechanisms (98). In support of a role for eosinophils, eosinophil count was also significantly correlated with control of mycobacterial growth in whole blood from African buffalo (102).

##### 3.4.3.4 Toll-like receptors

Toll-like receptors (TLRs) play an important role in innate immunity through the recognition of pathogen-associated molecular patterns (PAMPs), leading to recruitment of adaptor proteins which initiate signal transduction pathways culminating in the regulation of cytokine, chemokine and type I IFN expression. In the African buffalo study, TLR6 diversity was positively correlated with mycobacterial growth (ie. a higher proportion of heterozygous sites was associated with reduced ability to control mycobacterial growth) (102). The authors hypothesise that TLR6 diversity may act to improve recognition and macrophage activation at the expense of bacterial growth in the initial stages of infection, or that TLR6 in African buffalo is under selective pressure from other endemic bacterial species present in the natural environment (102).

## 4 Discussion

This systematic review has identified, evaluated and summarised available primary literature on the development and application of the direct MGIA. It is clear that the assay has seen significant progress over the past decade, with new descriptions in different species and assay standardisation and harmonisation efforts with formal assessments of reproducibility at multiple levels from intra-assay precision to inter-site repeatability. With increasing confidence from the field, it has seen exciting new applications to the evaluation of the specific and non-specific effects of BCG as well as the assessment of novel TB vaccine candidates, clinical cohorts, and an improved understanding of potential correlates of protective immunity. One of the advantages of the assay is its relative simplicity which makes it readily transferable, including to resource-limited settings.

Functional assays based on host cells with a bacterial target are notoriously challenging and a sensitive, reproducible, transferable MGIA, cannot be built in a day. Indeed, the now widely-used malaria GIA, simpler in its measurement of the host antibody response alone, took decades of collective development (1). One notable challenge is the lack of appropriate samples with which to optimise and validate the assay in humans. Given the requirement for a relatively high cell input and limitations on bleed volumes at any given time-point, comprehensively comparing assay performance over a range of co-culture conditions using the same human sample set is unfeasible. In the absence of a protective TB vaccine, BCG vaccination in populations where it is most efficacious (for example in the UK where it confers ~80% efficacy) is the best available option, although pre-existing baseline responses and heterogeneity in vaccine effect between individuals may still limit sensitivity to detect group differences, as illustrated by Joosten et al. (47). Satisfactory further optimisation of the human direct PBMC or whole blood MGIAs may necessitate a leukophoresis study in a large sample of healthy UK individuals pre- and post-BCG vaccination. The consistently high level of protection conferred by BCG vaccination in mice and higher number of cells obtained from a single spleen has permitted more extensive assay optimisation in this species, making it the most advanced and reliable of the direct MGIAs at the time of writing.

One must also be realistic about the extent to which a two-dimensional *in vitro* system measuring a response in one compartment at a single point in time can model the dynamic complexities of *in vivo* immunity. The expectation should not be for such an assay to perfectly recapitulate, and thus replace the need for, *in vivo* protection studies, but rather to provide a reasonable predictive surrogate that may be used as an early down-select, a complementary tool and/or a tractable model for mechanistic interrogation. Nonetheless, for the field to have confidence in using the assay to inform decisions about pursuing therapeutic or vaccine candidates, robust validation data should be generated and made available from multiple groups. Further assessment of assay performance relative to outcomes from *in vivo* challenge experiments for vaccine candidates conferring different levels of efficacy should thus be a priority.

While assay validation against *in vivo* protection from TB is possible in mice at the group level, and indeed this has been reported in several studies (65, 66), this is more challenging in humans due to the lack of TB controlled human infection models. Nonetheless, an association has been demonstrated between direct MGIA outcome and protection from experimental challenge with attenuated *M. bovis*, including following vaccination with MVA85A (56). NHPs are perhaps the best model for assay validation, offering as they do the opportunity to compare assay outcomes with protection from *in vivo M.tb* challenge on an individual animal basis (as animals are not culled to obtain cells as in the case of mice). The NHP direct MGIA also has the greatest 3Rs implications. However, limitations around sample availability and small group sizes due to economic and ethical considerations, cell viability following freeze-thawing and increased heterogeneity in responses compared with mice can also make it challenging to work with this species, necessitating further efforts before *in vivo* challenge experiments can be replaced with confidence.

Regarding the quality of the studies described, the majority were rated as ‘good’ or ‘very good’, although almost half failed to describe the methods in sufficient detail to allow replication. Another priority going forward should thus be the more comprehensive description of assay methods, which would support further assay transfer and uptake, as well as standardisation between groups. The lack of a suitable standardised quality assessment tool necessitated adaptation from two existing tools to define a combination of criteria best suited to the aims of the review. However, inclusion of aspects relating to interventions meant that studies without this design may have been disadvantaged by receiving an ‘NA’ (worth 0 points) for the intervention, randomisation and blinding categories. The development of validated quality assessment tools for *ex vivo* studies is needed.

The field should also aim for improved assay standardisation, both within and between species, to facilitate direct comparisons between studies and crucially between vaccine candidates tested at different institutes. Such a need for further optimisation and standardisation was identified as a key recommendation in a recent systematic review and meta-analysis of whole blood MGIAs (115). Optimal co-culture conditions for the murine assay consistent with the human and NHP iterations have recently been defined in a move towards aligning direct MGIA protocols and generating a cross-species consensus (116). Transferability to different species is a major advantage of the direct MGIA, and the development of direct MGIAs for other widely-used preclinical models of TB such as guinea-pigs, as well as wildlife species of relevance such as the European badger and wild boar, would be beneficial. The tractability of the human assay also provides opportunities to explore additional clinical cohorts, and work is underway to evaluate mycobacterial control in the context of conditions as broad-ranging as malaria and systemic lupus erythematous (SLE) (unpublished data).

While other GIAs may be based on single immune parameters, it is clear that control of mycobacterial growth in the direct MGIA is influenced by a range of innate and adaptive parameters that combine, likely synergistically, in a multifaceted picture. This is perhaps unsurprising given the complexity of the *in vivo* immune response to mycobacteria and failure after over a century of research to identify a validated immune correlate of protection from TB. As such, the direct MGIA represents, to at least some extent, the complex *in vivo* situation, which is indeed central to its aims and strengths. An assay outcome driven purely by IFN-γ producing T cells or trained innate immunity for example would add little to the tool-kit, while a functional assay that can recapitulate a more comprehensive ‘overview’ of the immune response (whether vaccine- or clinically-induced) offers a valuable complementary approach. Continued efforts to exploit the tractable nature of the assay in defining the relative contributions of different immune components to the control of mycobacterial growth are also highly informative and open new avenues of research for potential correlates of protection for TB.

In summary, we have provided an overview of the current literature on the development and range of applications of the direct MGIA since its conception. Risk of meta-biases is our review was reduced by predefining eligibility criteria, using broad and inclusive search terms, searching three independent databases, two authors independently screening search returns, and using additional citation screening to identify missed eligible papers. However, there are some limitations such as the exclusion of studies that used the direct MGIA method for the most part but a different mycobacterial quantification system; given the close correlation between BACTEC MGIT and agar plating outcomes, results from such studies would likely be informative and contribute to the body of literature. Furthermore, the diversity in study design, methodology, populations and sample sizes in the included studies hampered our ability to draw direct comparisons: a challenge also noted by Bok et al. in their review of whole blood MGIAs (115). Nonetheless, our synthesis of direct MGIA studies to date highlights the progress made over the past two decades and suggests that the assay may provide a valuable tool for the early evaluation of TB drug and vaccine candidates, clinical cohorts, and immune mechanisms of mycobacterial control.

## Data availability statement

The raw data supporting the conclusions of this article will be made available by the authors, without undue reservation.

## Author contributions

HP: Data curation, Formal analysis, Investigation, Methodology, Validation, Writing – original draft, Writing – review & editing. EH: Conceptualization, Methodology, Software, Writing – original draft, Writing – review & editing. HF: Supervision, Validation, Writing – original draft, Writing – review & editing. HM: Supervision, Validation, Writing – original draft, Writing – review & editing. RT: Conceptualization, Data curation, Formal analysis, Investigation, Methodology, Supervision, Validation, Visualization, Writing – original draft, Writing – review & editing.

## References

[1] Duncan CJ Hill AV Ellis RD . Can growth inhibition assays (GIA) predict blood-stage malaria vaccine efficacy? Hum Vaccin Immunother (2012) 8(6):706–14. doi: 10.4161/hv.19712 PMC3495712 22508415

[2] Naarding MA Fernandez N Kappes JC Hayes P Ahmed T Icyuz M . Development of a luciferase based viral inhibition assay to evaluate vaccine induced CD8 T-cell responses. J Immunol Methods (2014) 409:161–73. doi: 10.1016/j.jim.2013.11.021 PMC4236027 24291126

[3] Bash MC Lynn F Mocca B Borrow R Findlow H Hassan-King M . Development and use of a serum bactericidal assay using pooled human complement to assess responses to a meningococcal group A conjugate vaccine in African toddlers. Clin Vaccine Immunol (2014) 21(5):755–61. doi: 10.1128/CVI.00812-13 PMC4018891 24671551

[4] Tanner R McShane H . Replacing, reducing and refining the use of animals in tuberculosis vaccine research. ALTEX (2016) 34(1):157–66. doi: 10.14573/altex.1607281 27667476

[5] Kampmann B Gaora PO Snewin VA Gares MP Young DB Levin M . Evaluation of human antimycobacterial immunity using recombinant reporter mycobacteria. J Infect Dis (2000) 182(3):895–901. doi: 10.1086/315766 10950786

[6] Newton S Martineau A Kampmann B . A functional whole blood assay to measure viability of mycobacteria, using reporter-gene tagged BCG or M.Tb (BCGlux/M.Tb lux). J Vis Exp (2011) (55):3332. doi: 10.3791/3332-v 21946922 PMC3230178

[7] Silver RF Li Q Boom WH Ellner JJ . Lymphocyte-dependent inhibition of growth of virulent Mycobacterium tuberculosis H37Rv within human monocytes: requirement for CD4+ T cells in purified protein derivative-positive, but not in purified protein derivative-negative subjects. J Immunol (1998) 160(5):2408–17. doi: 10.4049/jimmunol.160.5.2408 9498784

[8] Hoft DF Worku S Kampmann B Whalen CC Ellner JJ Hirsch CS . Investigation of the relationships between immune-mediated inhibition of mycobacterial growth and other potential surrogate markers of protective Mycobacterium tuberculosis immunity. J Infect Dis (2002) 186(10):1448–57. doi: 10.1086/344359 12404160

[9] Parra M Yang AL Lim J Kolibab K Derrick S Cadieux N . Development of a murine mycobacterial growth inhibition assay for evaluating vaccines against Mycobacterium tuberculosis. Clin Vaccine Immunol (2009) 16(7):1025–32. doi: 10.1128/CVI.00067-09 PMC2708400 19458207

[10] Kolibab K Parra M Yang AL Perera LP Derrick SC Morris SL . A practical in *vitro* growth inhibition assay for the evaluation of TB vaccines. Vaccine (2009) 28(2):317–22. doi: 10.1016/j.vaccine.2009.10.047 19879231

[11] Carpenter E Fray L Gormley E . Cellular responses and Mycobacterium bovis BCG growth inhibition by bovine lymphocytes. Immunol Cell Biol (1997) 75(6):554–60. doi: 10.1038/icb.1997.86 9492191

[12] Denis M Wedlock DN Buddle BM . Ability of T cell subsets and their soluble mediators to modulate the replication of Mycobacterium bovis in bovine macrophages. Cell Immunol (2004) 232(1-2):1–8. doi: 10.1016/j.cellimm.2005.01.003 15922710

[13] Tanner R O’Shea MK Fletcher HA McShane H . *In vitro* mycobacterial growth inhibition assays: A tool for the assessment of protective immunity and evaluation of tuberculosis vaccine efficacy. Vaccine (2016) 10:2983. doi: 10.1016/j.vaccine.2016.07.058 27527814

[14] Brennan MJ Tanner R Morris S Scriba TJ Achkar JM Zelmer A . The cross-species mycobacterial growth inhibition assay (MGIA) project, 2010-2014. Clin Vaccine Immunol (2017) 24(9):e00142-17. doi: 10.1128/CVI.00142-17 28701467 PMC5585695

[15] Schlichter JG Mac Lean H . A method of determining the effective therapeutic level in the treatment of subacute bacterial endocarditis with penicillin; a preliminary report. Am Heart J (1947) 34(2):209–11. doi: 10.1016/0002-8703(47)90289-5 20255264

[16] Wallis RS Palaci M Vinhas S Hise AG Ribeiro FC Landen K . A whole blood bactericidal assay for tuberculosis. J Infect Dis (2001) 183(8):1300–3. doi: 10.1086/319679 11262217

[17] Page MJ McKenzie JE Bossuyt PM Boutron I Hoffmann TC Mulrow CD . The PRISMA 2020 statement: an updated guideline for reporting systematic reviews. BMJ (2021) 372:n71. doi: 10.1136/bmj.n71 33782057 PMC8005924

[18] Falconer J . Removing duplicates from an EndNote library. (2018). Available at: https://blogs.lshtm.ac.uk/library/2018/12/07/removing-duplicates-from-an-endnote-library/

[19] Ouzzani M Hammady H Fedorowicz Z Elmagarmid A . Rayyan—a web and mobile app for systematic reviews. Syst Rev (2016) 5:1–10. doi: 10.1186/s13643-016-0384-4 27919275 PMC5139140

[20] National Heart, L. a. B. I . Quality assessment tool for observational cohort and cross-sectional studies. (2014). Available at: https://www.nhlbi.nih.gov/health-topics/study-quality-assessment-tools

[21] Sheth VH Shah NP Jain R Bhanushali N Bhatnagar V . Development and validation of a risk-of-bias tool for assessing in *vitro* studies conducted in dentistry: The QUIN. J Prosthet Dent (2022) 22:S0022-3913(22)00345-6. doi: 10.1016/j.prosdent.2022.05.019 35752496

[22] Wallis RS Vinhas SA Johnson JL Ribeiro FC Palaci M Peres RL . Whole blood bactericidal activity during treatment of pulmonary tuberculosis. J Infect Dis (2003) 187(2):270–8. doi: 10.1086/346053 12552451

[23] Janulionis E Sofer C Schwander SK Nevels D Kreiswirth B Shashkina E . Survival and replication of clinical Mycobacterium tuberculosis isolates in the context of human innate immunity. Infect Immun (2005) 73(5):2595–601. doi: 10.1128/IAI.73.5.2595-2601.2005 PMC1087323 15845461

[24] Wallis RS Vinhas S Janulionis E . Strain specificity of antimycobacterial immunity in whole blood culture after cure of tuberculosis. Tuberculosis (2009) 89(3):221–4. doi: 10.1016/j.tube.2009.02.001 PMC2752959 19321387

[25] Wallis RS Ginindza S Beattie T Arjun N Likoti M Sebe M . Lung and blood early biomarkers for host-directed tuberculosis therapies: Secondary outcome measures from a randomized controlled trial. PloS One (2022) 17(2):e0252097. doi: 10.1371/journal.pone.0252097 35120127 PMC8815935

[26] Janulionis E Sofer C Song HY Wallis RS . Lack of activity of orally administered clofazimine against intracellular Mycobacterium tuberculosis in whole-blood culture. Antimicrob Agents Chemother (2004) 48(8):3133–5. doi: 10.1128/AAC.48.8.3133-3135.2004 PMC478499 15273133

[27] Wallis RS Jakubiec WM Kumar V Silvia AM Paige D Dimitrova D . Pharmacokinetics and Whole-Blood Bactericidal Activity against Mycobacterium tuberculosis of Single Doses of PNU-100480 in Healthy Volunteers. J Infect Dis (2010) 202(5):745–51. doi: 10.1086/655471 20629533

[28] Wallis RS Dawson R Friedrich SO Venter A Paige D Zhu T . Mycobactericidal activity of sutezolid (PNU-100480) in sputum (EBA) and blood (WBA) of patients with pulmonary tuberculosis. PloS One (2014) 9(4):e94462. doi: 10.1371/journal.pone.0094462 24732289 PMC3986205

[29] Wallis RS Good CE O’Riordan MA Blumer JL Jacobs MR Griffiss JM . Mycobactericidal activity of bedaquiline plus rifabutin or rifampin in ex vivo whole blood cultures of healthy volunteers: A randomized controlled trial. PloS One (2018) 13(5):e0196756. doi: 10.1371/journal.pone.0196756 29718967 PMC5931679

[30] Wallis RS Jakubiec W Kumar V Bedarida G Silvia A Paige D . Biomarker-assisted dose selection for safety and efficacy in early development of PNU-100480 for tuberculosis. Antimicrob Agents Chemother (2011) 55(2):567–74. doi: 10.1128/AAC.01179-10 PMC3028776 21078950

[31] Gurumurthy M Verma R Naftalin CM Hee KH Lu Q Tan KH . Activity of faropenem with and without rifampicin against Mycobacterium tuberculosis: evaluation in a whole-blood bactericidal activity trial. J Antimicrob Chemother (2017) 72(7):2012–9. doi: 10.1093/jac/dkx081 28333342

[32] Zhu T Friedrich SO Diacon A Wallis RS . Population pharmacokinetic/pharmacodynamic analysis of the bactericidal activities of sutezolid (PNU-100480) and its major metabolite against intracellular Mycobacterium tuberculosis in ex vivo whole-blood cultures of patients with pulmonary tuberculosis. Antimicrob Agents Chemother (2014) 58(6):3306–11. doi: 10.1128/AAC.01920-13 PMC4068491 24687496

[33] Naftalin Claire M Verma R Gurumurthy M Lu Q Zimmerman M Yeo Benjamin Chaik M . Coadministration of allopurinol to increase antimycobacterial efficacy of pyrazinamide as evaluated in a whole-blood bactericidal activity model. Antimicrob Agents Chemother (2017) 61(10):e00482-17. doi: 10.1128/aac.00482-00417 28739782 PMC5610504

[34] Naftalin CM Verma R Gurumurthy M Hee KH Lu Q Yeo BCM . Adjunctive use of celecoxib with anti-tuberculosis drugs: evaluation in a whole-blood bactericidal activity model. Sci Rep (2018) 8(1):13491. doi: 10.1038/s41598-018-31590-4 30202030 PMC6131161

[35] Verma R Gurumurthy M Yeo BCM Lu Q Naftalin CM Paton NI . Effects of increasing concentrations of rifampicin on different mycobacterium tuberculosis lineages in a whole-blood bactericidal activity assay. Antimicrob Agents Chemother (2022) 66(2):e0169921. doi: 10.1128/aac.01699-21 34871090 PMC8846316

[36] Saliu OY Sofer C Stein DS Schwander SK Wallis RS . Tumor-necrosis-factor blockers: differential effects on mycobacterial immunity. J Infect Dis (2006) 194(4):486–92. doi: 10.1086/505430 16845632

[37] Harausz EP Chervenak KA Good CE Jacobs MR Wallis RS Sanchez-Felix M . Activity of nitazoxanide and tizoxanide against Mycobacterium tuberculosis in *vitro* and in whole blood culture. Tuberculosis (2016) 98:92–6. doi: 10.1016/j.tube.2016.03.002 PMC4864490 27156623

[38] Wallis RS Jakubiec W Mitton-Fry M Ladutko L Campbell S . Rapid evaluation in whole blood culture of regimens for XDR-TB containing PNU-100480 (sutezolid), TMC207, PA-824, SQ109, and pyrazinamide. PLoS One (2012) 7(1):e30479. doi: 10.1371/journal.pone.0030479 22279595 PMC3261206

[39] Kwan PKW Lin W Naim ANM Periaswamy B De Sessions PF Hibberd ML . Gene expression responses to anti-tuberculous drugs in a whole blood model. BMC Microbiol (2020) 20(1):81. doi: 10.1186/s12866-020-01766-y 32264819 PMC7140558

[40] Reddy VM Dubuisson T Einck L Wallis RS Jakubiec W Ladukto L . SQ109 and PNU-100480 interact to kill Mycobacterium tuberculosis in *vitro* . J Antimicrob Chemother (2012) 67(5):1163–6. doi: 10.1093/jac/dkr589 22258923

[41] Wallis RS Song H-Y Whalen C Okwera A . TB chemotherapy. Am J Respir Crit Care Med (2004) 169(6):771–2. doi: 10.1164/ajrccm.169.6.954 15003953

[42] Cheon SH Kampmann B Hise AG Phillips M Song HY Landen K . Bactericidal activity in whole blood as a potential surrogate marker of immunity after vaccination against tuberculosis. Clin Diagn Lab Immunol (2002) 9(4):901–7. doi: 10.1128/CDLI.9.4.901-907.2002 PMC120034 12093693

[43] Fletcher HA Tanner R Wallis RS Meyer J Manjaly ZR Harris S . Inhibition of mycobacterial growth in *vitro* following primary but not secondary vaccination with Mycobacterium bovis BCG. Clin Vaccine Immunol (2013) 20(11):1683–9. doi: 10.1128/CVI.00427-13 PMC3837779 23986316

[44] Tuomela M Stanescu I Krohn K . Validation overview of bio-analytical methods. Gene Ther (2005) 12 Suppl 1:S131–8. doi: 10.1038/sj.gt.3302627 16231045

[45] Lee H Kim J Kang YA Kim DR Sim B Zelmer A . *In vitro* mycobacterial growth inhibition in South Korean adults with latent TB infection. Front Immunol (2019) 10:896–6. doi: 10.3389/fimmu.2019.00896 PMC6497970 31105706

[46] Smith SG Zelmer A Blitz R Fletcher HA Dockrell HM . Polyfunctional CD4 T-cells correlate with in *vitro* mycobacterial growth inhibition following Mycobacterium bovis BCG-vaccination of infants. Vaccine (2016) 34(44):5298–305. doi: 10.1016/j.vaccine.2016.09.002 27622301

[47] Joosten SA Meijgaarden KEV Arend SM Prins C Oftung F Korsvold GE . Mycobacterial growth inhibition is associated with trained innate immunity. J Clin Invest (2018) 128(5):1837–51. doi: 10.1172/JCI97508 PMC5919803 29461976

[48] Prabowo SA Smith SG Seifert K Fletcher HA . Impact of individual-level factors on Ex vivo mycobacterial growth inhibition: Associations of immune cell phenotype, cytomegalovirus-specific response and sex with immunity following BCG vaccination in humans. Tuberculosis (2019) 119:101876. doi: 10.1016/j.tube.2019.101876 31698310

[49] Prabowo SA Zelmer A Stockdale L Ojha U Smith SG Seifert K . Historical BCG vaccination combined with drug treatment enhances inhibition of mycobacterial growth ex vivo in human peripheral blood cells. Sci Rep (2019) 9(1):4842. doi: 10.1038/s41598-019-41008-4 30890730 PMC6425030

[50] Tanner R Smith SG van Meijgaarden KE Giannoni F Wilkie M Gabriele L . Optimisation, harmonisation and standardisation of the direct mycobacterial growth inhibition assay using cryopreserved human peripheral blood mononuclear cells. J Immunol Methods (2019) 469:1–10. doi: 10.1016/j.jim.2019.01.006 PMC7926177 30710562

[51] Tanner R Hoogkamer E Bitencourt J White A Boot C Sombroek CC . The in *vitro* direct mycobacterial growth inhibition assay (MGIA) for the early evaluation of TB vaccine candidates and assessment of protective immunity: a protocol for non-human primate cells. F1000Res (2021) 10:257. doi: 10.12688/f1000research.51640.1 33976866 PMC8097740

[52] Bitencourt J Peralta-Álvarez MP Wilkie M Jacobs A Wright D Salman Almujri S . Induction of functional specific antibodies, igG-secreting plasmablasts and memory B cells following BCG vaccination. Front Immunol (2021) 12:798207. doi: 10.3389/fimmu.2021.798207 35069580 PMC8767055

[53] Harris SA Meyer J Satti I Marsay L Poulton ID Tanner R . Evaluation of a human BCG challenge model to assess antimycobacterial immunity induced by BCG and a candidate tuberculosis vaccine, MVA85A, alone and in combination. J Infect Dis (2014) 209(8):1259–68. doi: 10.1093/infdis/jit647 PMC3969545 24273174

[54] Minassian AM Satti I Poulton ID Meyer J Hill AV McShane H . A human challenge model for Mycobacterium tuberculosis using Mycobacterium bovis bacille Calmette-Guerin. J Infect Dis (2012) 205(7):1035–42. doi: 10.1093/infdis/jis012 PMC3295601 22396610

[55] Minhinnick A Harris S Wilkie M Peter J Stockdale L Manjaly-Thomas ZR . Optimization of a human bacille calmette-guérin challenge model: A tool to evaluate antimycobacterial immunity. J Infect Dis (2016) 213(5):824–30. doi: 10.1093/infdis/jiv482 PMC4747614 26450421

[56] Tanner R Satti I Harris SA O’Shea MK Cizmeci D O’Connor D . Tools for Assessing the Protective Efficacy of TB Vaccines in Humans: in *vitro* Mycobacterial Growth Inhibition Predicts Outcome of in *vivo* Mycobacterial Infection. Front Immunol (2020) 10:2983. doi: 10.3389/fimmu.2019.02983 31998295 PMC6968127

[57] Stylianou E Paul MJ Reljic R McShane H . Mucosal delivery of tuberculosis vaccines: a review of current approaches and challenges. Expert Rev Vaccines (2019) 18(12):1271–84. doi: 10.1080/14760584.2019.1692657 PMC6961305 31876199

[58] Dijkman K Sombroek CC Vervenne RAW Hofman SO Boot C Remarque EJ . Prevention of tuberculosis infection and disease by local BCG in repeatedly exposed rhesus macaques. Nat Med (2019) 25(2):255–62. doi: 10.1038/s41591-018-0319-9 30664782

[59] White AD Sarfas C Sibley LS Gullick J Clark S Rayner E . Protective efficacy of inhaled BCG vaccination against ultra-low dose aerosol M. tuberculosis challenge in rhesus macaques. Pharmaceutics (2020) 12(5):394. doi: 10.3390/pharmaceutics12050394 32344890 PMC7284565

[60] Darrah PA Zeppa JJ Maiello P Hackney JA Wadsworth MH Hughes TK . Prevention of tuberculosis in macaques after intravenous BCG immunization. Nature (2020) 577(7788):95–102. doi: 10.1038/s41586-019-1817-8 31894150 PMC7015856

[61] Radloff J Heyckendorf J van der Merwe L Sanchez Carballo P Reiling N Richter E . Mycobacterium growth inhibition assay of human alveolar macrophages as a correlate of immune protection following mycobacterium bovis bacille calmette–guérin vaccination. Front Immunol (2018) 9:1708. doi: 10.3389/fimmu.2018.01708 30087678 PMC6066571

[62] Andersson B Nordvall MJ Welin A Lerm M Schön T . “A novel mycobacterial growth inhibition assay employing live-cell imaging of virulent M. tuberculosis and monitoring of host cell viability. Tuberculosis (2020) 124:101977. doi: 10.1016/j.tube.2020.101977 32829078

[63] Marsay L Matsumiya M Tanner R Poyntz H Griffiths KL Stylianou E . “Mycobacterial growth inhibition in murine splenocytes as a surrogate for protection against Mycobacterium tuberculosis (M. tb). Tuberculosis (Edinb) (2013) 93(5):551–7. doi: 10.1016/j.tube.2013.04.007 23726784

[64] Zelmer A Tanner R Stylianou E Damelang T Morris S Izzo A . A new tool for tuberculosis vaccine screening: Ex vivo Mycobacterial Growth Inhibition Assay indicates BCG-mediated protection in a murine model of tuberculosis. BMC Infect Dis (2016) 16:412. doi: 10.1186/s12879-016-1751-4 27519524 PMC4983071

[65] Yang AL Schmidt TE Stibitz S Derrick SC Morris SL Parra M . A simplified mycobacterial growth inhibition assay (MGIA) using direct infection of mouse splenocytes and the MGIT system. J Microbiol Methods (2016) 131:7–9. doi: 10.1016/j.mimet.2016.09.010 27650198

[66] Jensen C Lindebo Holm L Svensson E Aagaard C Ruhwald M . Optimisation of a murine splenocyte mycobacterial growth inhibition assay using virulent Mycobacterium tuberculosis. Sci Rep (2017) 7(1):2830. doi: 10.1038/s41598-017-02116-1 28588268 PMC5460210

[67] Painter H Prabowo SA Cia F Stockdale L Tanner R Willcocks S . Adaption of the ex vivo mycobacterial growth inhibition assay for use with murine lung cells. Sci Rep (2020) 10(1):3311. doi: 10.1038/s41598-020-60223-y 32094451 PMC7039920

[68] Flynn JL Gideon HP Mattila JT Lin PL . Immunology studies in non-human primate models of tuberculosis. Immunol Rev (2015) 264(1):60–73. doi: 10.1111/imr.12258 25703552 PMC4339213

[69] Laddy DJ Bonavia A Hanekom WA Kaushal D Williams A Roederer M . Toward tuberculosis vaccine development: recommendations for nonhuman primate study design. Infect Immun (2018) 86(2):e00776-17. doi: 10.1128/IAI.00776-17 29203540 PMC5778361

[70] Tanner R White AD Boot C Sombroek CC O’Shea MK Wright D . A non-human primate in *vitro* functional assay for the early evaluation of TB vaccine candidates. NPJ Vaccines (2021) 6(1):3. doi: 10.1038/s41541-020-00263-7 33397986 PMC7782578

[71] Sibley L White AD Gooch KE Stevens LM Tanner R Jacobs A . High-dose Mycobacterium tuberculosis aerosol challenge cannot overcome BCG-induced protection in chinese origin cynomolgus macaques; implications of natural resistance for vaccine evaluation. Sci Rep (2021) 11(1):12274. doi: 10.1038/s41598-021-90913-0 34112845 PMC8192909

[72] Waters WR Palmer MV Buddle BM Vordermeier HM . Bovine tuberculosis vaccine research: Historical perspectives and recent advances. Vaccine (2012) 30(16):2611–22. doi: 10.1016/j.vaccine.2012.02.018 22342705

[73] Srinivasan S Conlan AJK Easterling LA Herrera C Dandapat P Veerasami M . A meta-Analysis of the effect of Bacillus Calmette-Guérin vaccination against bovine tuberculosis: is perfect the enemy of good? Front Vet Sci (2021) 8:637580. doi: 10.3389/fvets.2021.637580 33681334 PMC7930010

[74] Waters WR Palmer MV Thacker TC Davis WC Sreevatsan S Coussens P . Tuberculosis immunity: opportunities from studies with cattle. Clin Dev Immunol (2011) 2011:768542. doi: 10.1155/2011/768542 21197095 PMC3004413

[75] Pepponi I Khatri B Tanner R Villarreal-Ramos B Vordermeier M McShane H . A mycobacterial growth inhibition assay (MGIA) for bovine TB vaccine development. Tuberculosis (Edinb) (2017) 106:118–22. doi: 10.1016/j.tube.2017.07.008 PMC5670120 28802398

[76] de Bree LCJ Koeken VACM Joosten LAB Aaby P Benn CS van Crevel R . Non-specific effects of vaccines: Current evidence and potential implications. Semin Immunol (2018) 39:35–43. doi: 10.1016/j.smim.2018.06.002 30007489

[77] Uthayakumar D Paris S Chapat L Freyburger L Poulet H De Luca K . Non-specific effects of vaccines illustrated through the BCG example: from observations to demonstrations. Front Immunol (2018) 9:2869. doi: 10.3389/fimmu.2018.02869 30564249 PMC6288394

[78] Moorlag S Arts RJW Van Crevel R Netea MG . Non-specific effects of BCG vaccine on viral infections. Clin Microbiol Infect (2019) 25(12):1473–8. doi: 10.1016/j.cmi.2019.04.020 31055165

[79] Wilkie M Tanner R Wright D Lopez Ramon R Beglov J Riste M . Functional *in-vitro* evaluation of the non-specific effects of BCG vaccination in a randomised controlled clinical study. Sci Rep (2022) 12(1):7808. doi: 10.1038/s41598-022-11748-x 35552463 PMC9096342

[80] Alexandroff AB Jackson AM O’Donnell MA James K . BCG immunotherapy of bladder cancer: 20 years on. Lancet (1999) 353(9165):1689–94. doi: 10.1016/S0140-6736(98)07422-4 10335805

[81] Bilsen MP van Meijgaarden KE de Jong HK Joosten SA Prins C Kroft LJM . A novel view on the pathogenesis of complications after intravesical BCG for bladder cancer. Int J Infect Dis (2018) 72:63–8. doi: 10.1016/j.ijid.2018.05.006 29778583

[82] Pujilestari R Rukmana A Karuniawati A . Efficacy of Tuberculosis Vaccine Candidate pcDNA3. 1-rpfB in Inhibiting the Growth of Mycobacterium tuberculosis *In Vitro* with Mycobacterial Growth Inhibition Assay. Makara J Sci (2022) 26(1):6. doi: 10.7454/mss.v26i1.1260

[83] Nurfadilah M Rukmana A Sjatha F . “Evaluation of Tuberculosis Vaccine Candidate, pcDNA3. 1-rpfD using Mycobacterial Growth Inhibition Assay (MGIA). HAYATI J Biosci (2022) 29(1):1–8. doi: 10.4308/hjb.29.1.1-8

[84] Tanner R O’Shea MK White AD Müller J Harrington-Kandt R Matsumiya M . The influence of haemoglobin and iron on in *vitro* mycobacterial growth inhibition assays. Sci Rep (2017) 7:43478. doi: 10.1038/srep43478 28256545 PMC5335253

[85] Hoft DF Blazevic A Selimovic A Turan A Tennant J Abate G . Safety and immunogenicity of the recombinant BCG vaccine AERAS-422 in healthy BCG-naïve adults: A randomized, active-controlled, first-in-human phase 1 trial. EBioMedicine (2016) 7:278–86. doi: 10.1016/j.ebiom.2016.04.010 PMC4909487 27322481

[86] Sagawa ZK Goman C Frevol A Blazevic A Tennant J Fisher B . Safety and immunogenicity of a thermostable ID93 + GLA-SE tuberculosis vaccine candidate in healthy adults. Nat Commun (2023) 14(1):1138. doi: 10.1038/s41467-023-36789-2 36878897 PMC9988862

[87] Prabowo SA Painter H Zelmer A Smith SG Seifert K Amat M . RUTI Vaccination Enhances Inhibition of Mycobacterial Growth ex vivo and Induces a Shift of Monocyte Phenotype in Mice. Front Immunol (2019) 10:894–4. doi: 10.3389/fimmu.2019.00894 PMC6503078 31114572

[88] O’Shea MK Tanner R Müller J Harris SA Wright D Stockdale L . Immunological correlates of mycobacterial growth inhibition describe a spectrum of tuberculosis infection. Sci Rep (2018) 8(1):14480. doi: 10.1038/s41598-018-32755-x 30262883 PMC6160428

[89] Dijkman K Vervenne RAW Sombroek CC Boot C Hofman SO van Meijgaarden KE . Disparate tuberculosis disease development in macaque species is associated with innate immunity. Front Immunol (2019) 10:2479–9. doi: 10.3389/fimmu.2019.02479 PMC6838139 31736945

[90] Baguma R Penn-Nicholson A Smit E Erasmus M Day J Makhethe L . Application of a whole blood mycobacterial growth inhibition assay to study immunity against Mycobacterium tuberculosis in a high tuberculosis burden population. PloS One (2017) 12(9):e0184563–e0184563. doi: 10.1371/journal.pone.0184563 28886145 PMC5590973

[91] Venkataraman A Shanmugam S Balaji S Mani K Shanmugavel AK Muthuramalingam K . Comparison of two mycobacterial strains in performance of the whole blood mycobacterial growth inhibition assay in Indian children. Tuberculosis (2022) 137:102255. doi: 10.1016/j.tube.2022.102255 36252397

[92] du Plessis N Walzl G . Helminth-M. tb co-infection,” How helminths alter immunity to infection. (New York, NY: Springer) (2014). pp. 49–74. 10.1007/978-1-4939-1489-0_3 25253027

[93] Salgame P Yap GS Gause WC . Effect of helminth-induced immunity on infections with microbial pathogens. Nat Immunol (2013) 14(11):1118–26. doi: 10.1038/ni.2736 PMC4955540 24145791

[94] Maizels RM McSorley HJ . Regulation of the host immune system by helminth parasites. J Allergy Clin Immunol (2016) 138(3):666–75. doi: 10.1016/j.jaci.2016.07.007 PMC5010150 27476889

[95] Resende Co T Hirsch CS Toossi Z Dietze R Ribeiro-Rodrigues R . Intestinal helminth co-infection has a negative impact on both anti-Mycobacterium tuberculosis immunity and clinical response to tuberculosis therapy. Clin Exp Immunol (2007) 147(1):45–52. doi: 10.1111/j.1365-2249.2006.03247.x 17177962 PMC1810442

[96] Elias D Wolday D Akuffo H Petros B Bronner U Britton S . Effect of deworming on human T cell responses to mycobacterial antigens in helminth-exposed individuals before and after bacille Calmette–Guérin (BCG) vaccination. Clin Exp Immunol (2001) 123(2):219–25. doi: 10.1046/j.1365-2249.2001.01446.x PMC1905995 11207651

[97] Potian JA Rafi W Bhatt K McBride A Gause WC Salgame P . Preexisting helminth infection induces inhibition of innate pulmonary anti-tuberculosis defense by engaging the IL-4 receptor pathway. J Exp Med (2011) 208(9):1863–74. doi: 10.1084/jem.20091473 PMC3171086 21825018

[98] O’Shea MK Fletcher TE Muller J Tanner R Matsumiya M Bailey JW . Human hookworm infection enhances mycobacterial growth inhibition and associates with reduced risk of tuberculosis infection. Front Immunol (2018) 9:2893–3. doi: 10.3389/fimmu.2018.02893 PMC6302045 30619265

[99] Bobadilla-del-Valle M Leal-Vega F Torres-Gonzalez P Ordaz-Vazquez A Garcia-Garcia M d. L . Mycobacterial Growth Inhibition Assay (MGIA) as a host directed diagnostic tool for the evaluation of the immune response in subjects living with type 2 diabetes mellitus. Front Cell Infect Microbiol (2021) 11:640707. doi: 10.3389/fcimb.2021.640707 34084753 PMC8167894

[100] Kewcharoenwong C Prabowo SA Bancroft GJ Fletcher HA Lertmemongkolchai G . Glibenclamide reduces primary human monocyte functions against tuberculosis infection by enhancing M2 polarization. Front Immunol (2018) 9:2109. doi: 10.3389/fimmu.2018.02109 30283449 PMC6157405

[101] Naranbhai V Fletcher H Tanner R O’Shea M McShane H Fairfax B . Distinct transcriptional and anti-mycobacterial profiles of peripheral blood monocytes dependent on the ratio of monocytes: lymphocytes. EBioMedicine (2015) 2:1619–26. doi: 10.1016/j.ebiom.2015.09.027 PMC4740301 26870787

[102] le Roex N Jolles A Beechler B van Helden P Hoal E . Toll-like receptor (TLR) diversity influences mycobacterial growth in African buffalo. Tuberculosis (Edinb) (2017) 104:87–94. doi: 10.1016/j.tube.2017.03.009 28454655

[103] Tanner R Villarreal-Ramos B Vordermeier HM McShane H . The humoral immune response to BCG vaccination. Front Immunol (2019) 10:1317. doi: 10.3389/fimmu.2019.01317 31244856 PMC6579862

[104] Kawahara JY Irvine EB Alter G . A case for antibodies as mechanistic correlates of immunity in tuberculosis. Front Immunol (2019) 10:996–6. doi: 10.3389/fimmu.2019.00996 PMC6521799 31143177

[105] Chen T Blanc C Eder AZ Prados-Rosales R Souza AC Kim RS . Association of human antibodies to arabinomannan with enhanced mycobacterial opsonophagocytosis and intracellular growth reduction. J Infect Dis (2016) 214(2):300–10. doi: 10.1093/infdis/jiw141 PMC4918826 27056953

[106] Pahari S Kaur G Negi S Aqdas M Das DK Bashir H . Reinforcing the functionality of mononuclear phagocyte system to control tuberculosis. Front Immunol (2018) 9:193. doi: 10.3389/fimmu.2018.00193 29479353 PMC5811511

[107] Cross GB Yeo BC Hutchinson PE Tan MC Verma R Lu Q . Impact of selective immune-cell depletion on growth of Mycobacterium tuberculosis (Mtb) in a whole-blood bactericidal activity (WBA) assay. PloS One (2019) 14(5):e0216616. doi: 10.1371/journal.pone.0216616 31100071 PMC6524797

[108] Naranbhai V Hill AV Abdool Karim SS Naidoo K Abdool Karim Q Warimwe GM . Ratio of monocytes to lymphocytes in peripheral blood identifies adults at risk of incident tuberculosis among HIV-infected adults initiating antiretroviral therapy. J Infect Dis (2014) 209(4):500–9. doi: 10.1093/infdis/jit494 PMC3903371 24041796

[109] Naranbhai V Kim S Fletcher H Cotton MF Violari A Mitchell C . The association between the ratio of monocytes:lymphocytes at age 3 months and risk of tuberculosis (TB) in the first two years of life. BMC Med (2014) 12:120. doi: 10.1186/s12916-014-0120-7 25034889 PMC4223414

[110] Naranbhai V Moodley D Chipato T Stranix-Chibanda L Nakabaiito C Kamateeka M . The association between the ratio of monocytes: lymphocytes and risk of tuberculosis among HIV-infected postpartum women. J Acquir Immune Defic Syndr (2014) 67(5):573–5. doi: 10.1097/QAI.0000000000000353 PMC4229408 25247435

[111] McNeill E Stylianou E Crabtree MJ Harrington-Kandt R Kolb A-L Diotallevi M . Regulation of mycobacterial infection by macrophage Gch1 and tetrahydrobiopterin. Nat Commun (2018) 9(1):5409. doi: 10.1038/s41467-018-07714-9 30573728 PMC6302098

[112] Wu C Li Z Fu X Yu S Lao S Yang B . Antigen-specific human NKT cells from tuberculosis patients produce IL-21 to help B cells for the production of immunoglobulins. Oncotarget (2015) 6(30):28633–45. doi: 10.18632/oncotarget.5764 PMC4745682 26416419

[113] Pulido D Torrent M Andreu D Nogués MV Boix E . Two human host defense ribonucleases against mycobacteria, the eosinophil cationic protein (RNase 3) and RNase 7. Antimicrob Agents Chemother (2013) 57(8):3797–805. doi: 10.1128/AAC.00428-13 PMC3719706 23716047

[114] Driss V Legrand F Hermann E Loiseau S Guerardel Y Kremer L . TLR2-dependent eosinophil interactions with mycobacteria: role of α-defensins. Blood J Am Soc Hematol (2009) 113(14):3235–44. doi: 10.1182/blood-2008-07-166595 18978205

[115] Bok J Hofland RW Evans CA . Whole blood mycobacterial growth assays for assessing human tuberculosis susceptibility: A systematic review and meta-analysis. Front Immunol (2021) 12:641082. doi: 10.3389/fimmu.2021.641082 34046032 PMC8144701

[116] Tanner R Zelmer A Painter H Stylianou E Pinpathomrat N Harrington-Kandt R . Assessment of the reproducibility and inter-site transferability of the murine direct splenocyte mycobacterial growth inhibition assay (MGIA). bioRxiv (2021), 2021.2002.2014.431105. doi: 10.1101/2021.02.14.431105

