# Supplementary material for: Development and application of the direct mycobacterial growth inhibition assay: a systematic review
Source: Front Immunol. 2024 Feb 6;15:1355983. doi: 10.3389/fimmu.2024.1355983 (PMC10877019; doi:10.3389/fimmu.2024.1355983)
Supplement: Supplementary file 1 [file DataSheet_1.docx]

**SUPPLEMENTARY INFORMATION**

PubMed

(((((((((((("Biological Assay"[Mesh:NoExp]) OR "In Vitro Techniques"[Mesh:NoExp]) OR "Culture Techniques"[Mesh:NoExp]) OR "Cell Culture Techniques"[Mesh:NoExp]) OR "Primary Cell Culture"[Mesh:NoExp]) OR "Lung"[Mesh:NoExp]) OR "Humans"[Mesh]) OR "Mice"[Mesh:NoExp]) OR "Macaca"[Mesh:NoExp]) OR "Cattle"[Mesh:NoExp]) OR (assay*[Title/Abstract] OR "in vitro"[Title/Abstract] OR "whole blood"[Title/Abstract] OR PBMC*[Title/Abstract] OR "peripheral blood mononuclear cells"[Title/Abstract] OR splenocyte*[Title/Abstract] OR lung*[Title/Abstract] OR pulmonary[Title/Abstract] OR human*[Title/Abstract] OR patient*[Title/Abstract] OR subject*[Title/Abstract] OR participant*[Title/Abstract] OR case[Title/Abstract] OR cases[Title/Abstract] OR mouse[Title/Abstract] OR mice[Title/Abstract] OR murine[Title/Abstract] OR "non-human primate*"[Title/Abstract] OR "nonhuman primate*"[Title/Abstract] OR NHP*[Title/Abstract] OR cattle[Title/Abstract] OR bovine[Title/Abstract] OR cow*[Title/Abstract])) AND ("growth inhibition"[Text Word] OR "mycobacterial immunity"[Text Word] OR "antimycobacterial immunity"[Text Word] OR MGIA[Text Word] OR bactericid*[Text Word] OR "inhibiting growth"[Text Word] OR "anti-mycobacterial immunity"[Text Word])) AND ((("Mycobacterium"[Mesh]) OR "Tuberculosis"[Mesh]) OR (mycobacterial[Title/Abstract] OR mycobacteria[Title/Abstract] OR mycobacterium[Title/Abstract] OR tuberculosis[Title/Abstract] OR BCG[Title/Abstract] OR TB[Title/Abstract])) Filters: English, from 2001 – 2023

Embase

Database: Embase 1974 to present

Search Strategy:

1 bioassay/ (58858)

2 in vitro study/ (1519095)

3 cell culture technique/ (6083)

4 primary cell culture / (5551)

5 lung/ (193727)

6 exp human/ (25440585)

7 mouse/ (2107987)

8 Macaca/ (23621)

9 bovine/ (50207)

10 (assay* or bioassay* or "in vitro" or "whole blood" or PBMC* or "peripheral blood mononuclear cells" or splenocyte* or lung* or pulmonary or human* or patient* or subject* or participant* or case or cases or mouse or mice or murine or "non-human primate*" or "nonhuman primate*" or NHP* or cattle or bovine or cow*).ti,ab. (21712818)

11 1 or 2 or 3 or 4 or 5 or 6 or 7 or 8 or 9 or 10 (30985380)

12 growth inhibition/ (62466)

13 ("growth inhibition" or "mycobacterial immunity" or "antimycobacterial immunity" or MGIA or bactericid* or "inhibiting growth" or "anti-mycobacterial immunity").ti,ab. (96456)

14 12 or 13 (137545)

15 exp Mycobacterium/ (127608)

16 exp tuberculosis/ (222702)

17 (mycobacterial or mycobacteria or mycobacterium or tuberculosis or BCG or TB).ti,ab. (300860)

18 15 or 16 or 17 (372644)

19 11 and 14 and 18 (3822)

20 19 (3822)

21 limit 20 to (english language and yr="2001 -Current") (2930)

SCOPUS

( TITLE-ABS-KEY ( assay* OR bioassay* OR "in vitro" OR "whole blood" OR pbmc* OR "peripheral blood mononuclear cells" OR splenocyte* OR lung* OR pulmonary OR human* OR patient* OR subject* OR participant* OR case OR cases OR mouse OR mice OR murine OR "non-human primate*" OR "nonhuman primate*" OR nhp* OR cattle OR bovine OR cow* ) AND TITLE-ABS-KEY ( "growth inhibition" OR "mycobacterial immunity" OR "antimycobacterial immunity" OR mgia OR bactericid* OR "inhibiting growth" OR "anti-mycobacterial immunity" ) AND TITLE-ABS-KEY ( mycobacterial OR mycobacteria OR mycobacterium OR tuberculosis OR bcg OR tb ) ) AND ( LIMIT-TO ( PUBYEAR , 2023 ) OR LIMIT-TO ( PUBYEAR , 2022 ) OR LIMIT-TO ( PUBYEAR , 2021 ) OR LIMIT-TO ( PUBYEAR , 2020 ) OR LIMIT-TO ( PUBYEAR , 2019 ) OR LIMIT-TO ( PUBYEAR , 2018 ) OR LIMIT-TO ( PUBYEAR , 2017 ) OR LIMIT-TO ( PUBYEAR , 2016 ) OR LIMIT-TO ( PUBYEAR , 2015 ) OR LIMIT-TO ( PUBYEAR , 2014 ) OR LIMIT-TO ( PUBYEAR , 2013 ) OR LIMIT-TO ( PUBYEAR , 2012 ) OR LIMIT-TO ( PUBYEAR , 2011 ) OR LIMIT-TO ( PUBYEAR , 2010 ) OR LIMIT-TO ( PUBYEAR , 2009 ) OR LIMIT-TO ( PUBYEAR , 2008 ) OR LIMIT-TO ( PUBYEAR , 2007 ) OR LIMIT-TO ( PUBYEAR , 2006 ) OR LIMIT-TO ( PUBYEAR , 2005 ) OR LIMIT-TO ( PUBYEAR , 2004 ) OR LIMIT-TO ( PUBYEAR , 2003 ) OR LIMIT-TO ( PUBYEAR , 2002 ) OR LIMIT-TO ( PUBYEAR , 2001 ) ) AND ( LIMIT-TO ( LANGUAGE , "English" ) )

**Supplementary Table 1. Search strategies**

| **Title and reference number** | **Year** | **Journal** | **Authors** | **Species** | **Cohort** | **Sample size** | **Intervention(s)** | **Sample type (eg. WB, PBMC, splenocytes)** | **WB/Cell input** | **Mycobacterial strain and inoculum** | **Co-culture volume and time period** | **Other immune parameters measured** | **Are they assessed for correlation with control of mycobacterial growth?** |
| --- | --- | --- | --- | --- | --- | --- | --- | --- | --- | --- | --- | --- | --- |
| A whole blood bactericidal assay for tuberculosis [16] | 2001 | *Journal of Infectious Diseases* | Wallis, R. S. and Palaci, M. and Vinhas, S. and Hise, A. G. and Ribeiro, F. C. and Landen, K. and Cheon, S. H. and Song, H. Y. and Phillips, M. and Dietze, R. and Elner, J. J. | Human | One healthy, tuberculin skin test–negative volunteer + two healthy volunteers | 1 + 2 | Rifampin (600 mg), isoniazid (300 mg), levofloxacin (750 mg), moxifloxacin (400 mg), pyrazinamide (25 mg/kg), ethambutol (25 mg/kg), amoxicillin/clavulanate (750 mg), levofloxacin/pyrazinamide/ethambutol (as per individual drugs), isoniazid/pyrazinamide/rifampin | WB (collected before and at intervals following drug administration) | 0.3 ml WB | MDR and sensitive M. tb isolates (~10^4^ CFU) | 0.6 ml; 72 hours | No | NR |
| Whole blood bactericidal activity during treatment of pulmonary tuberculosis [22] | 2003 | *Journal of Infectious Diseases* | Wallis, R. S. and Vinhas, S. A. and Johnson, J. L. and Ribeiro, F. C. and Palaci, M. and Peres, R. L. and Sa, R. T. and Dietze, R. and Chiunda, A. and Eisenach, K. and Ellner, J. J. | Human | HIV-1-seronegative patients 18–60 years old with newly diagnosed initial episodes of sputum smear-positive pulmonary TB | 36 | Isoniazid, rifampin, ethambutol and pyrazinamide, daily for 60 days, followed by isoniazid and rifampin, daily for 120 days; WBA performed at 8-, 12- and 28-weeks post treatment initiation; drug doses 300, 300, 600, and 1000 mg (weight <35 kg), 300, 450, 800 and 1500 mg (weight 35–45 kg), and 400, 600, 1200, and 2000 mg (weight >45 kg). | WB (collected before and at intervals following drug administration) | 0.3 ml WB | H37Ra, MPO-28, patient isolate; 5x10^4^-1x10^5^ CFU | 0.6 ml; 72 hours | No | NR |
| Survival and replication of clinical *Mycobacterium tuberculosis* isolates in the context of human innate immunity [23] | 2005 | *Infection and Immunity* | Janulionis, E. and Sofer, C. and Schwander, S. K. and Nevels, D. and Kreiswirth, B. and Shashkina, E. and Wallis, R. S. | Human | Healthy PPD–negative volunteers | 6 | None | WB | 0.3 ml WB | 18 clinical isolates, H37Rv, H37Ra, HN878, CDC1551, MP-28; volume predicted to be positive in 4.5 days | 0.6 ml; 24 and 72 hours | Strain-specific kinetics; Protein microarray: IL-1β, TNF, TNFRII, ICAM, MCP-1, MIP-1α, IL-10, TGF-β1 and IL-12 p40; ELISA: IFNα and IFNγ | No correlation between growth in broth and growth in WB cultures; inverse correlation between log growth rate and interquartile range i.e., more differences among subjects for attenuated versus virulent strains |
| Strain specificity of antimycobacterial immunity in whole blood culture after cure of tuberculosis [24] | 2009 | *Tuberculosis (Edinb)* | Wallis, R. S. and Vinhas, S. and Janulionis, E. | Human | 18–60-year-old, HIV-1-negative individuals with cured pulmonary TB and healthy TST-negative volunteers | 32 + 6 | None | WB | 0.3 ml WB | H37Ra, MPO-28 and patient isolates; inoculum not specified | 0.6 ml; 72 hours | No | NR |
| Lung and blood early biomarkers for host-directed tuberculosis therapies: Secondary outcome measures from a randomized controlled trial [25] | 2022 | *PLoS ONE* | Wallis, R. S. and Ginindza, S. and Beattie, T. and Arjun, N. and Likoti, M. and Sebe, M. and Edward, V. A. and Rassool, M. and Ahmed, K. and Fielding, K. and Ahidjo, B. A. and Vangu, M. D. T. and Churchyard, G. | Human | Adults with Cepheid Xpert MTB/RIF sputum testing showing rifampin-susceptible M. tb, and ≥1 probe showing a cycle threshold <20; moderately advanced or far advanced pulmonary tuberculosis by chest radiograph; body weight 40-90 kg | 200 | Patients received CC-11050 200 mg BID; everolimus 0.5 mg QD; auranofin 6 mg QD after an initial week of 3 mg QD; ergocalciferol 5 mg on day 1, then 2.5mg on days 28 and 56; or control; all patients additionally received standard tuberculosis treatment with rifabutin 300 mg QD substituted for rifampin | WB | 0.3 ml WB obtained on study day 42 (intensive phase), day 84 (continuation phase), and day 140 (after experimental treatments were complete) | H37Rv; volume predicted to be positive in 5.5 days | 0.6 ml; 72 hours | No | NR |
| Lack of activity of orally administered clofazimine against intracellular *Mycobacterium tuberculosis* in whole-blood culture [26] | 2004 | *Antimicrobial Agents and Chemotherapy* | Janulionis, E. and Sofer, C. and Song, H. Y. and Wallis, R. S. | Human | Healthy tuberculin-nonreactive volunteers | 10 | Daily ofloxacin (600 mg) or clofazimine (200 mg) for five days; pyrazinamide (25 mg/kg) added on days 6-10; ethambutol (25 mg/kg) added on day 10. | WB | 0.3 ml WB | H37Rv; inoculum not specified | 0.6 ml; 72 hours | No | NR |
| Pharmacokinetics and whole-blood bactericidal activity against *Mycobacterium tuberculosis* of single doses of PNU-100480 in healthy volunteers [27] | 2010 | *Journal of Infectious Diseases* | Wallis, R. S. and Jakubiec, W. M. and Kumar, V. and Silvia, A. M. and Paige, D. and Dimitrova, D. and Li, X. and Ladutko, L. and Campbell, S. and Friedland, G. and Mitton-Fry, M. and Miller, P. F. | Human | Healthy subjects | 19 + 8 | Up to two escalating single oral doses of PNU-100480 (35, 100, 300, 600, 1000, or 1500 mg) or placebo, or linezolid once daily for 4 days (300 mg) | WB obtained 0, 2, 3, 6, 12, and 24 hours post PNU-100480, or 12 hours after third dose and at 0, 2, 3, 4, and 8 hours after fourth dose of linezolid | 0.3 ml WB | H37Rv; volume predicted to be positive in 5.5 days | 0.6 ml; 72 hours | No | NR |
| Mycobactericidal activity of sutezolid (PNU-100480) in sputum (EBA) and blood (WBA) of patients with pulmonary tuberculosis [28] | 2014 | *PLoS ONE* | Wallis, R. S. and Dawson, R. and Friedrich, S. O. and Venter, A. and Paige, D. and Zhu, T. and Silvia, A. and Gobey, J. and Ellery, C. and Zhang, Y. and Eisenach, K. and Miller, P. and Diacon, A. H. | Human | 18–65-year-old sputum smear positive TB patients, HIV negative and positive | 59 | Sutezolid (600 mg BID) or 1,200 mg QD or fixed dose combination Rifafour | WB collected prior to treatment on day 1, 8 and 12 hours post dose on day 13 and at 0, 1, 2, 3, and 6 hours post dose on day 14 | 0.3 ml WB | H37Rv; predicted to be positive in 5.5 days | 0.6 ml; 72 hours | Sputum bactericidal activity | No relationship shown |
| Mycobactericidal activity of bedaquiline plus rifabutin or rifampin in *ex vivo* whole blood cultures of healthy volunteers: A randomized controlled trial [29] | 2018 | *PLoS ONE* | Wallis, R. S. and Good, C. E. and O'Riordan, M. A. and Blumer, J. L. and Jacobs, M. R. and McLeod Griffiss, J. and Healan, A. and Salata, R. A. | Human | Healthy adults | 33 | 400 mg oral bedaquiline (days 1 and 29) and either 600 mg oral rifampin or 300 mg rifabutin (days 20–41) | WB | 0.3 ml WB collected immediately prior to bedaquiline dosing and at 1, 2-, 3-, 4-, 6-, 8-, and 12-hours post-dose | H37Rv; volume predicted to be positive in 5.5 days | 0.6 ml; 72 hours | Pharmacokinetic analysis | Plasma concentration of 355, 120 and 22 ng/ml of bedaquiline, rifampin and rifabutin respectively required for intracellular mycobacteriostasis |
| Biomarker-assisted dose selection for safety and efficacy in early development of PNU-100480 for tuberculosis [30] | 2011 | *Antimicrobial Agents and Chemotherapy* | Wallis, R. S. and Jakubiec, W. and Kumar, V. and Bedarida, G. and Silvia, A. and Paige, D. and Zhu, T. and Mitton-Fry, M. and Ladutko, L. and Campbell, S. and Miller, P. F. | Human | Healthy volunteers, ages 18 to 55 years | 58 | PNU-100480 (100, 300, or 600 mg BID and 1,200 mg QD) or placebo for 14 days; or PNU-100480 at 600 mg or placebo BID as 200-mg tablets at a 4:1 ratio for 28 days, plus pyrazinamide (25 mg/kg) on days 27 and 28; or linezolid 300 mg QD for 4 days | WB 0, 2, 3, 4, 8, and 12 hours postdosing | 0.3 ml WB | H37Rv; volume predicted to be positive in 5.5 days | 0.6 ml; 72 hours | PK analysis | MIC and AUC/MIC significantly correlated with cumulative WBA during dosing of PNU-100480 |
| Activity of faropenem with and without rifampicin against *Mycobacterium tuberculosis*: evaluation in a whole-blood bactericidal activity trial [31] | 2017 | *Journal of Antimicrobial Chemotherapy* | Gurumurthy, M. and Verma, R. and Naftalin, C. M. and Hee, K. H. and Lu, Q. and Tan, K. H. and Issac, S. and Lin, W. and Tan, A. and Seng, K. Y. and Lee, L. S. U. and Paton, N. I. | Human | Healthy, M. tb-uninfected adults | 36 | Single oral dose of faropenem (600 mg) with amoxicillin/clavulanic acid (500/125 mg), rifampicin (10 mg/kg) or the combination rifampicin + faropenem + amoxicillin/clavulanic acid. | WB collected pre-dose and at 0.5-, 1-, 2-, 3-, 4-, 5-, 6- and 8-hours post-dose | 0.3 ml WB | H37Rv; volume of mycobacteria predicted to be positive in 5.5 days | 0.8 ml; 72 hours | Drug stability; MIC90 and MBC99 | No relationship between WBA and faropenem plasma concentration; clear relationship between WBA and rifampicin plasma concentration |
| Population pharmacokinetic/pharmacodynamic analysis of the bactericidal activities of sutezolid (pnu-100480) and its major metabolite against intracellular *Mycobacterium tuberculosis* in *ex vivo* whole-blood cultures of patients with pulmonary tuberculosis [32] | 2014 | *Antimicrobial Agents and Chemotherapy* | Zhu, T. and Friedrich, S. O. and Diacon, A. and Wallis, R. S. | Human | 18- to 65-year-old men and women with pulmonary TB, HIV negative and positive | 59 | Sutezolid (600 mg BID) or 1200 mg QD or fixed dose combination Rifafour | WB collected at baseline and days 13 and 14 (0, 1, 2, 3, 6, 8 and 12-hours hours post dosing) | 0.3 ml WB | H37Rv; volume predicted to be positive in 5.5 days | 0.6 ml; 72 hours | No | NR |
| Coadministration of allopurinol to increase antimycobacterial efficacy of pyrazinamide as evaluated in a whole-blood bactericidal activity model [33] | 2017 | *Antimicrobial Agents and Chemotherapy* | Naftalin, C. M. and Verma, R. and Gurumurthy, M. and Lu, Q. and Zimmerman, M. and Yeo, B. C. M. and Tan, K. H. and Lin, W. and Yu, B. and Dartois, V. and Paton, N. I. | Human | Healthy, Asian, IGRA-negative volunteers | 12 | Single dose PZA alone (10 or 25 mg/kg), followed by a second dose of PZA 7 days later with allopurinol (four daily 100 mg doses, from two days prior to the day after PZA dosing). | WB | 0.3 ml WB | H37Rv; volume predicted to be positive in 5.5 days | 0.6 ml; 96 hours | Pharmacokinetic assays - pyrazinamide, pyrazinoic acid, 5-hydroxypyrazinamide, and 5-hydroxypyrazinoic acid levels in plasma | Significant association between higher pyrazinoic acid levels and greater activity in the WBA assay; no relationship between cumulative WBA and the AUCs of pyrazinoic acid and pyrazinamide |
| Adjunctive use of celecoxib with anti-tuberculosis drugs: evaluation in a whole-blood bactericidal activity model [34] | 2018 | *Scientific reports* | Naftalin, C. M. and Verma, R. and Gurumurthy, M. and Hee, K. H. and Lu, Q. and Yeo, B. C. M. and Tan, K. H. and Lin, W. and Yu, B. and Seng, K. Y. and Lee, L. S. U. and Paton, N. I. | Human | Healthy volunteers | 18 | Three visits to clinic days 0, 7, 14 - visit 1, 400 mg celecoxib single dose; visit 2, 10 mg/kg rifampicin or 25 mg/kg pyrazinamine; visit 3, celecoxib plus pyrazinamine or rifampicin | WB | 0.3 ml WB obtained pre-dose and at 1, 1.5, 2, 3, 4, 6, 8 hours post-dose | H37Rv; volume predicted to be positive in 5.5 days | 0.6 ml; 72 hours | Pharmacokinetic analysis | Bactericidal activity significantly higher with increasing plasma concentrations of rifampicin and pyrazinamide |
| Effects of increasing concentrations of rifampicin on different *Mycobacterium tuberculosis* lineages in a whole-blood bactericidal activity assay [35] | 2022 | *Antimicrobial Agents and Chemotherapy* | Verma, R. and Gurumurthy, M. and Yeo, B. C. M. and Lu, Q. and Naftalin, C. M. and Paton, N. I. | Human | Healthy adult volunteers | 8 | None | WB | 0.3 ml WB | Four clinical strains and a viable but conditionally non-replicating M. tb strain (streptomycin-starved M. tb 18b); 1x10^5^ CFU | 0.6 ml; 72 hours +/- 0.63-60 mg/l rifampicin | No | NR |
| Tumor-necrosis-factor blockers: differential effects on mycobacterial immunity [36] | 2006 | *Journal of Infectious Diseases* | Saliu, O. Y. and Sofer, C. and Stein, D. S. and Schwander, S. K. and Wallis, R. S. | Human | Healthy volunteers | 20 | None | WB | 0.3 ml WB | H37Ra; volume predicted to be positive in 4.5 days | 0.6 ml; 24 and 96 hours; -/+ adalimumab, infliximab and etanercept | No | NR |
| Activity of nitazoxanide and tizoxanide against *Mycobacterium tuberculosis* *in vitro* and in whole blood culture [37] | 2016 | *Tuberculosis* | Harausz, E. P. and Chervenak, K. A. and Good, C. E. and Jacobs, M. R. and Wallis, R. S. and Sanchez-Felix, M. and Boom, W. H. | Human | Healthy TST- or QFT-negative adults | 11 | None | WB | 0.5 ml WB + 2, 4, 16, 32, 64 or 128 µg/ml nitazoxanide or tizoxanide | 1x10^6^ H37Rv | Volume not specified; 72 hours | Co-culture of BCG with nitazoxanide or tizoxanide plus increasing concentrations of human plasma | In WB and plasma studies, 72-hour co-incubation resulted in loss of anti-mycobacterial activity of drugs. |
| Rapid evaluation in whole blood culture of regimens for XDR-TB containing PNU-100480 (sutezolid), TMC207, PA-824, SQ109, and pyrazinamide [38] | 2012 | *PLoS ONE* | Wallis, R. S. and Jakubiec, W. and Mitton-Fry, M. and Ladutko, L. and Campbell, S. and Paige, D. and Silvia, A. and Miller, P. F. | Human | Healthy donor | 1 | None | WB | 0.3 ml WB +/- TMC207, PNU-1004800, PNU-101603, PA-824, rifampin, isoniazid, pyrazinamide or dihydroxy-vitamin D | H37Rv; volume predicted to be positive in 5.5 days | 0.6 ml; 72 hours | Sputum bacillary count | Cumulative WBA greater in patients with drug-sensitive TB during intensive phase of standard therapy; correlated with rate of decline of sputum log CFU; superior in patients whose sputum cultures converted to negative after 8 weeks of treatment |
| Gene expression responses to anti-tuberculous drugs in a whole blood model [39] | 2020 | *BMC Microbiology* | Kwan, P. K. W. and Lin, W. and Naim, A. N. M. and Periaswamy, B. and De Sessions, P. F. and Hibberd, M. L. and Paton, N. I. | Human | Healthy adult volunteers or adult patients with drug-susceptible pulmonary TB | 3 + 4 + 9 (TB) | None | WB | 0.3 ml WB | H37Rv; volume predicted to be positive in 5.5 days | 0.6 ml; 0.5, 1 , 1.5, 2, 4, 6, 24, 48 and 72 hours +/- rifampicin 10 μg/ml, isoniazid 5 μg/ml, pyrazinamide 25μg/ml, ethambutol 5 μg/ml, moxifloxacin 4 μg/ml, faropenem sodium 5 μg/ml, amoxicillin 10 μg/ml or clavulanic acid 3 μg/ml | Gene expression profiling of WB cultures and sputum samples from TB patients | Agreement between WB cultures and sputum and ability to differentiate individual drugs indicates that transcriptomics may add value to the WBA for the evaluation of TB drugs |
| SQ109 and PNU-100480 interact to kill *Mycobacterium tuberculosis in vitro* [40] | 2012 | *Journal of Antimicrobial Chemotherapy* | Reddy, V. M. and Dubuisson, T. and Einck, L. and Wallis, R. S. and Jakubiec, W. and Ladukto, L. and Campbell, S. and Nacy, C. A. | Human | Healthy donors | 2 | None | WB | 0.3 ml WB +/- SQ109, PNU-100480 and PNU-101603 at fixed 1:5 ratio | H37Rv; inoculum not specified | 0.6 ml; 72 hours | No | NR |
| TB chemotherapy: antagonism between immunity and sterilization [41] | 2004 | *American Journal of Respiratory and Critical Care Medicine* | Wallis, R. S. and Song, H. Y. and Whalen, C. and Okwera, A. | Human | Healthy PPD–negative volunteers and patients with TB | Not specified | None | WB | 0.3 ml WB +/- ofloxacin | H37Rv; inoculum not specified | 0.6 ml; 72 hours | No | NR |
| Bactericidal activity in whole blood as a potential surrogate marker of immunity after vaccination against tuberculosis [42] | 2002 | *Clinical and Diagnostic Laboratory Immunology* | Cheon, S. H. and Kampmann, B. and Hise, A. G. and Phillips, M. and Song, H. Y. and Landen, K. and Li, Q. and Larkin, R. and Ellner, J. J. and Silver, R. F. and Hoft, D. F. and Wallis, R. S. | Human | PPD-positive and -negative adults | 10 + 6 | I.d. BCG Connaught vaccination (3x10^6 CFU); 8/10 individuals revaccinated at six months | WB | 0.3 ml WB | BCG, H37Ra, H37Rv, clinical isolate (MP-28) (~5x10^3^ CFU) | 0.6 ml; 0, 24, 72 and 96 hours | CD4^+^ and CD8^+^ T-cell depletion from WB; inhibition and quantification of TNFα by ELISA | Inhibition of TNFα resulted in increased growth of H37Ra in PPD-reactive donors; no effect in PPD non-reactors; no effect on growth of the clinical isolate; removal of T cells produced additive effects in the control of H37Ra but not the clinical isolate |
| Inhibition of mycobacterial growth *in vitro* following primary but not secondary vaccination with *Mycobacterium bovis* BCG [43] | 2013 | *Clinical and Vaccine Immunology* | Fletcher, H. A. and Tanner, R. and Wallis, R. S. and Meyer, J. and Manjaly, Z. R. and Harris, S. and Satti, I. and Richard, F. S. and Hoft, D. and Kampmann, B. and Walker, K. B. and Dockrell, H. M. and Fruth, U. and Barker, L. and Brennan, M. J. and McShane, H. | Human | Healthy adults with no history of BCG vaccination or a history of prior BCG vaccination more than six months prior to study enrolment | 18 (WB) + 19 (PBMC) | Single i.d. immunisation with BCG-SSI | WB and PBMC | 0.3 ml WB or 106 PBMCs | WB - 150 CFU BCG Pasteur; PBMC - 600 CFU BCG Pasteur | 0.6 ml; 96 hours | *Ex vivo* IFNγ ELISPOT assay on isolated PMBCs | Mycobacterial growth inhibition does not correlate with IFNγ ELISPOT responses |
| *In vitro* mycobacterial growth inhibition in South Korean adults with latent TB infection [45] | 2019 | *Frontiers in Immunology* | Lee, H. and Kim, J. and Kang, Y. A. and Kim, D. R. and Sim, B. and Zelmer, A. and Fletcher, H. A. and Dockrell, H. M. and Smith, S. G. and Cho, S. N. | Human | Healthy adults | 121 | None | PBMC | 1x10^6^ PBMCs | BCG Pasteur; 100 CFU | 0.6 ml; 96 hours | WB ICS and IGRA | Individuals who were QFT negative had improved ability to inhibit BCG growth; household TB contacts without known TB had improved ability to inhibit BCG growth vs those with latent TB infection and known TB contact; higher PPD-specific, polyfunctional T_H_1 responses in WB did not correlate with enhanced mycobacteria control in PBMC |
| Polyfunctional CD4 T-cells correlate with *in vitro* mycobacterial growth inhibition following *Mycobacterium bovis* BCG-vaccination of infants [46] | 2016 | *Vaccine* | Smith, S. G. and Zelmer, A. and Blitz, R. and Fletcher, H. A. and Dockrell, H. M. | Human | Healthy, UK-born infants, born to mothers with no history of chronic illness including HIV infection | 30 | Single dose of i.d. BCG SSI or unvaccinated | PBMCs from four months and one year post vaccination (time-matched in unvaccinated) | 1x10^6^ PBMCs | 862 CFU BCG Danish | 0.6 ml; 96 hours | WB ICS | Increased capacity to inhibit *in vitro* growth of mycobacteria in PBMC from BCG-vaccinated infants correlates with polyfunctional T-cell response. |
| Mycobacterial growth inhibition is associated with trained innate immunity [47] | 2018 | *Journal of Clinical Investigation* | Joosten, S. A. and Van Meijgaarden, K. E. and Arend, S. M. and Prins, C. and Oftung, F. and Korsvold, G. E. and Kik, S. V. and Arts, R. J. W. and Van Crevel, R. and Netea, M. G. and Ottenhoff, T. H. M. | Human | Four cohorts: i) - healthy donors, PPD negative or prior to BCG vaccination; ii) individuals with latent TB infection; iii) archived samples - contact investigations at a soccer club, in a supermarket and among immigrants with a recent TB contact; iv) healthy male volunteers | Sample size for cohorts i and ii unclear from text; 85 (cohort iii) + 30 (cohort iv) | Cohorts i and iv – BCG-/placebo-vaccinated | PBMCs | 1x10^6^ PBMCs; cohort i - samples obtained 4, 8, and 12 weeks and 1 year after BCG vaccination; cohort ii - samples obtained at study inclusion (month 0), 6 and 24 months later; cohort iii - soccer club samples from inclusion, 6, 12, 18 24 months later cohort iv -samples obtained before BCG/placebo and 4 weeks later | BCG; 2.6 log CFU | 0.6 ml; 96 hours | Stimulation with BCG for flow cytometry including ICS; qRT-PCR; 40-plex chemo-cytokine Luminex; overnight culture with H37Rv, heat- killed *Candida albicans* or *Staphylococcus aureus* | No positive correlation between absolute QuantiFERON result and control of BCG growth; %CD19^+^ B cells correlated with control of BCG growth in the MGIA; %CD3^+^ T cells inversely related to BCG growth in the MGIA; no correlation between frequency of CD8^+^ or CD4^+^ multifunctional T cells and MGIA results; CD4^+^ and CD8^+^ effector cell frequencies inversely related with MGIA control; %CD14^+^, ML ratio and CD14^dim^ monocytes correlated with control of mycobacterial outgrowth; non-classical monocytes produced CXCL10, and CXCR3 receptor blockade inhibited control of BCG outgrowth |
| Impact of individual-level factors on *ex vivo* mycobacterial growth inhibition: associations of immune cell phenotype, cytomegalovirus-specific response and sex with immunity following BCG vaccination in humans [48] | 2019 | *Tuberculosis* | Prabowo, S. A. and Smith, S. G. and Seifert, K. and Fletcher, H. A. | Human | Healthy adult participants with no history of BCG vaccination or a history of BCG vaccination >6 months before study enrolment | 100 | None | PBMCs | 3x10^6^ PBMCs | BCG Pasteur Aeras; 100 CFU | 0.6 ml; 96 hours | ELISA; IFNγ ELISpot; flow cytometric immune phenotyping and ICS flow cytometry | Inverse correlation between higher IFNγ ELISpot response and lower mycobacterial growth; correlation between IL-10 production and mycobacterial growth; no significant correlations between frequencies of BCG-specific CD4^+^ and CD8^+^ T-cells and mycobacterial growth; inverse correlation between frequency of NK cells and mycobacterial growth; T-cell activation correlated with mycobacterial growth |
| Historical BCG vaccination combined with drug treatment enhances inhibition of mycobacterial growth *ex vivo* in human peripheral blood cells [49] | 2019 | *Scientific reports* | Prabowo, S. A. and Zelmer, A. and Stockdale, L. and Ojha, U. and Smith, S. G. and Seifert, K. and Fletcher, H. A. | Human | Healthy adults with no evidence of exposure or infection with TB | 21 BCG-naïve and 29 BCG-vaccinated volunteers | None | PBMC | 3x10^6^ PBMCs | BCG Pasteur; 100 CFU | 0.6 ml; 96 hours +/- 1, 0.1, 0.01 isoniazid or 0.5, 0.1, 0.01 μg/ml rifampicin | IFNγ ELISpot response to PPD; ELISA; ICS and flow cytometry | No statistically significant correlation between IFNγ ELISpot response and mycobacterial growth; In the presence of 1 μg/ml isoniazid an inverse correlation between IFNγ production detected by ELISA and *ex vivo* growth inhibition and positive correlation between IL-10 production and higher growth of mycobacteria; In the presence of 0.1 μg/ml rifampicin a positive correlation between IL-10 production and higher growth of mycobacteria |
| Optimisation, harmonisation and standardisation of the direct mycobacterial growth inhibition assay using cryopreserved human peripheral blood mononuclear cells [50] | 2019 | *Journal of Immunological Methods* | Tanner, R. and Smith, S. G. and van Meijgaarden, K. E. and Giannoni, F. and Wilkie, M. and Gabriele, L. and Palma, C. and Dockrell, H. M. and Ottenhoff, T. H. M. and McShane, H. | Human | Healthy Dutch donors or healthy UK adults; both enrolled in BCG vaccination study | Sample size not specified | Single i.d. dose of 2-8x10^5^ CFU BCG SSI | PBMC | 1x10^6^ or 3x10^6^ PBMC; isolated from buffy coat or peripheral blood obtained pre-vaccination at days 0, and days 2, 4, 7, 10, 14, 21, 28 and 84 post-vaccination | BCG Pasteur; 500 CFU | 0.6 ml; 96 hours | Cell viability following 96-hour co-culture; IFNγ ELISA | No correlation between cell viability and mycobacterial growth in the MGIA; significant correlation between IFNγ concentration and cell viability; significant correlation between IFNγ concentration in MGIA supernatant and volunteer PPD status |
| The *in vitro* direct mycobacterial growth inhibition assay (MGIA) for the early evaluation of TB vaccine candidates and assessment of protective immunity: a protocol for non-human primate cells [51] | 2021 | *F1000Research* | Tanner, R. and Hoogkamer, E. and Bitencourt, J. and White, A. and Boot, C. and Sombroek, C. C. and Harris, S. A. and O'Shea, M. K. and Wright, D. and Wittenberg, R. and Sarfas, C. and Satti, I. and Verreck, F. A. W. and Sharpe, S. A. and Fletcher, H. A. and McShane, H. | Human and NHP | Human volunteers and Rhesus macaques | Not specified (human) + 7 (NHPs) | None | PBMC | 1x10^6^ (human) or 3x10^6^ (NHP) plus autologous serum or plasma matched to animal and time point | BCG Pasteur; 100 or 500 CFU | 0.6 ml; 96 hours | No | NR |
| Induction of functional specific antibodies, IgG-secreting plasmablasts and memory B Cells following BCG vaccination [52] | 2021 | *Frontiers in Immunology* | Bitencourt, J. and Peralta-Alvarez, M. P. and Wilkie, M. and Jacobs, A. and Wright, D. and Salman Almujri, S. and Li, S. and Harris, S. A. and Smith, S. G. and Elias, S. C. and White, A. D. and Satti, I. and Sharpe, S. S. and O'Shea, M. K. and McShane, H. and Tanner, R. | Human and macaques | Human cohort 1, healthy BCG-naïve UK adults; human cohort 2, adult male latent TB-negative BCG-naive military recruits recently arrived in the UK from Nepal and group of historically BCG-vaccinated individuals; macaque cohort, rhesus or cynomolgus | 35 + 15 + 6 (macaques) | Unvaccinated or single i.d. vaccination with 2-8x10^5 CFU BCG SSI (human cohort) or BCG Danish 1331 (macaque) | PMBCs | 3x10^6 PBMCs plus autologous serum | BCG Pasteur Aeras, 500 CFU | 0.6 ml; 96 hours | ELISA; antibody-secreting cell ELISpot; memory B-cell ELISpot | Serum factors contribute to BCG-induced control of mycobacterial growth *in vitro* |
| Evaluation of a human BCG challenge model to assess antimycobacterial immunity induced by BCG and a candidate tuberculosis vaccine, MVA85A, alone and in combination [53] | 2014 | *Journal of Infectious Diseases* | Harris, S. A. and Meyer, J. and Satti, I. and Marsay, L. and Poulton, I. D. and Tanner, R. and Minassian, A. M. and Fletcher, H. A. and McShane, H. | Human | 18–55-year-old healthy adults, BCG-naive and -vaccinated | 23 + 26 | MVA85A (1x 10^8^ PFU i.d.) followed by challenge with i.d. BCG SSI (2-8x10^5^ CFU i.d.) | WB obtained on the day of BCG challenge | 0.3 ml WB | BCG; inoculum not specified | 0.6 ml; 96 hours | BCG copy number; skin biopsy CFU; *ex vivo* IFNγ ELISpot | Non-significant positive correlation between growth ratio and BCG copy number by PCR and growth ratio and CFU |
| Tools for assessing the protective efficacy of TB vaccines in humans: *in vitro* mycobacterial growth inhibition predicts outcome of *in vivo* mycobacterial infection [56] | 2020 | *Frontiers in Immunology* | Tanner, R. and Satti, I. and Harris, S. A. and O'Shea, M. K. and Cizmeci, D. and O'Connor, D. and Chomka, A. and Matsumiya, M. and Wittenberg, R. and Minassian, A. M. and Meyer, J. and Fletcher, H. A. and McShane, H. | Human | Healthy UK adults, no latent TB infection +/- historical BCG vaccination; two cohorts | 11 + 43 | Cohort 1, i.d. challenge with 1-4x10^5^ CFU BCG SSI; cohort 2, i) BCG naive, no vaccine administered in study, ii) BCG naive, i.d. MVA85A 1x10^8^ PFU administered during study; iii) BCG vaccinated 8-38 years prior to enrolment, no vaccine administered in study, iv) BCG vaccinated 8-38 years prior to enrolment, i.d. MVA85A 1x10^8^ PFU administered during study, groups i-iv, i.d. challenge with 1-4x10^5^ CFU BCG SSI | PBMCs | 3x10^6^ PBMCs plus autologous serum or plasma | BCG Pasteur Aeras, 500 CFU | 0.6 ml; 96 hours | Quantification of BCG load in skin biopsy; Intracellular cytokine staining; *ex vivo* IFNγ ELISpot; ELISA; gene expression microarray analysis | Positive correlation between MGIA mycobacterial growth and BCG recovered from biopsies in the BCG-vaccinated group by qPCR and CFU; control of MGIA mycobacterial growth is associated with IFNγ ELISpot response, and frequencies of PPD-specific IFNγ or TNFα producing CD4^+^ T cells and specific subpopulations of polyfunctional CD4^+^ T cells; transcription enrichment of gene sets for antigen processing/presentation and the IL-23 pathway associated with good controllers of mycobacterial growth; enrichment for hypoxia-related pathways in poor controllers |
| Mycobacterium growth inhibition assay of human alveolar macrophages as a correlate of immune protection following *Mycobacterium bovis* Bacille calmette-guerin vaccination [61] | 2018 | *Frontiers in Immunology* | Radloff, J. and Heyckendorf, J. and van der Merwe, L. and Carballo, P. S. and Reiling, N. and Richter, E. and Lange, C. and Kalsdorf, B. | Human | Healthy, BCG-naive subjects | 17 | Intracutaneous BCG vaccination | PBMCs and BALCs | 1x10^6 PBMCs or BALCs obtained before and eight weeks following vaccination | H37Rv; 5.8x10^4^ CFU | 0.6 ml; 96 hours +/- vitamin D3 | Surface and ICS of PBMC and BALCs; IGRA | Magnitude of PPD-response induced by BCG-vaccination did not correlate with growth control in BALC and PBMC |
| A novel mycobacterial growth inhibition assay employing live-cell imaging of virulent M. tuberculosis and monitoring of host cell viability [62] | 2020 | *Tuberculosis* | Andersson, B. and Nordvall, M. J. and Welin, A. and Lerm, M. and Schon, T. | Human | Swedish healthy blood donors | Not specified | None | PBMC | 1x10^5^ | GFP- or mCherry-expressing H37Rv; 5 , 0.1 and 0.01 MOI in relation to monocyte fraction of PBMC | 0.2 ml; 120 hours | Cell viability; imaging flow cytometry for detection of phagocytosis; fluorescence vs TTP in BACTEC MGIT system | PMBC viability higher using lower MOI; fluorescence measurements showed a high positive correlation between RFU and CFU above 100 bacteria |
| Mycobacterial growth inhibition in murine splenocytes as a surrogate for protection against *Mycobacterium tuberculosis* (M.tb) [63] | 2013 | *Tuberculosis (Edinb)* | Marsay, L. and Matsumiya, M. and Tanner, R. and Poyntz, H. and Griffiths, K. L. and Stylianou, E. and Marsh, P. D. and Williams, A. and Sharpe, S. and Fletcher, H. and McShane, H. | Mouse | 6–8-week-old female C57BL/6 mice | 8-10/group | Mice immunised with 1x10^6^ CFU BCG Pasteur s.c. at the base of the tail and then rested for 6 weeks | Splenocytes | 1x10^6^ splenocytes | BCG Pasteur; volume predicted to be positive in 6.5 days | Volume not specified; 96 hours | M. tb aerosol challenge; microarray analysis of splenocytes from mice used in MGIA (12 h co-culture with BCG [1:1]) | Mycobacterial growth inhibition by splenocytes from BCG-vaccinated mice corresponds with BCG-mediated protection from M. tb challenge; differential gene expression in splenocytes from BCG-vaccinated vs control mice (T_H_1 responses, including IFNγ, NOS2 and IL-17) |
| A new tool for tuberculosis vaccine screening: *ex vivo* mycobacterial growth inhibition assay indicates BCG-mediated protection in a murine model of tuberculosis [64] | 2016 | *BMC Infectious Diseases* | Zelmer, A. and Tanner, R. and Stylianou, E. and Damelang, T. and Morris, S. and Izzo, A. and Williams, A. and Sharpe, S. and Pepponi, I. and Walker, B. and Hokey, D. A. and McShane, H. and Brennan, M. and Fletcher, H. | Mouse | 5-7-week-old female C57BL/6 and B6.129S7-Ifngtm1Ts/J (IFNγ-/-) mice |  | s.c. injection of 100 μl BCG Pasteur Aeras or SSI or physiological saline solution into leg flap | Splenocytes harvested six weeks post vaccination | 1-5x10^6^ splenocytes | 90-3800 CFU BCG Pasteur Aeras or SSI; 100 CFU M. smegmatis | 0.6 ml; 96 hours | *In vivo* experimental infection with Erdman | BCG SSI and Pasteur Aeras vaccination conferred protection against experimental infection with Erdman in the lung and spleen; growth inhibition in the MGIA was observed following vaccination with BCG Pasteur Aeras only |
| A simplified mycobacterial growth inhibition assay (MGIA) using direct infection of mouse splenocytes and the MGIT system [65] | 2016 | *Journal of Microbiological Methods* | Yang, A. L. and Schmidt, T. E. and Stibitz, S. and Derrick, S. C. and Morris, S. L. and Parra, M. | Mouse |  | 6 | BCG, BCG formulated in DDA/TDB adjuvant, or the ESAT6-antigen 85B (SD1) fusion protein suspended in DDA/TDB adjuvant | Splenocytes obtained six weeks post vaccination | 5x10^6^ splenocytes; groups pooled to generate five replicates | 500 CFU M. tb | Volume not specified; 96 hours | *In vivo* experimental infection with M. tb | Significant correlation between *in vivo* experimental infection and MGIA data |
| Optimisation of a murine splenocyte mycobacterial growth inhibition assay using virulent *Mycobacterium tuberculosis* [66] | 2017 | *Scientific reports* | Jensen, C. and Lindebo Holm, L. and Svensson, E. and Aagaard, C. and Ruhwald, M. | Mouse | 6–8-week-old female CB6F1 mice | 3-8 mice/group | s.c. vaccination in base of tail three times at two-week intervals with Tris HCL buffer, CAF01 (dose 250 μg/50 μg (DDA/TDB)) alone or CAF01 mixed with 5 μg H56 protein, or single dose 1x10^6^ BCG Danish 1331 or single dose 1x106 BCG Danish 1331 followed by 0.1 μg H56 in CAF01 the next day, followed by two H56:CAF01 immunisations, two-weeks apart | Splenocytes harvested one week post final vaccination | 5x10^6^ splenocytes | Erdman; 25, 50, 250, 500 CFU | 0.6 ml standard media or enriched media | Intracellular cytokine staining of splenocytes; multiplex cytokine assay on splenocyte MGIA supernatants | No detectable infection driven expansion of vaccine-specific CD4+ T cell populations during the four-day culture; significant inverse correlation between IFNγ release and log_10_ CFU, no difference in IL-6 or -10 |
| Adaption of the *ex vivo* mycobacterial growth inhibition assay for use with murine lung cells [67] | 2020 | *Scientific reports* | Painter, H. and Prabowo, S. A. and Cia, F. and Stockdale, L. and Tanner, R. and Willcocks, S. and Reljic, R. and Fletcher, H. A. and Zelmer, A. | Mouse | 5–7-week-old female C57BL/6 mice | 6 mice/group (pooled to generate technical replicates, 4/group) | s.c. vaccination with 1x10^6^ CFU BCG Pasteur Aeras; i.n. vaccination with 0.5x10^6^ CFU BCG Pasteur Aeras or spore-FP1 | Lung and spleen cells | 1-3x10^6^ cells | BCG Pasteur Aeras or Erdman; 100 or 200 CFU respectively | 0.6 ml; 96 hours | No | NR |
| A non-human primate *in vitro* functional assay for the early evaluation of TB vaccine candidates [70] | 2021 | *npj Vaccines* | Tanner, R. and White, A. D. and Boot, C. and Sombroek, C. C. and O'Shea, M. K. and Wright, D. and Hoogkamer, E. and Bitencourt, J. and Harris, S. A. and Sarfas, C. and Wittenberg, R. and Satti, I. and Fletcher, H. A. and Verreck, F. A. W. and Sharpe, S. A. and McShane, H. | NHP | Cynomolgus and rhesus macaques | 67 | Study 1: Animals received 2-8 x10^5^ CFU BCG Danish i.d., followed by challenge with 1-4x10^6^ CFU BCG Danish i.d. 21 weeks post vaccination. Study 2: animals received 2-8x10^5^ CFU BCG Danish i.d., followed by challenge with 2-8x10^6^ CFU BCG Danish i.d.. Study 3: animals received 2-8 x10^5^ CFU BCG Danish i.d., followed by endobronchial instillation of 500 CFU Erdman K01 38 weeks post vaccination. Study 4, animals were unvaccinated, or received 2-8x10^5^ CFU BCG i.d., 2-8x10^6^ CFU i.v. or 2-8x10^5^ CFU BCG i.d. followed by 2-8x10^6^ CFU BCG i.t. 12 weeks later (study 4). All animals in study 4 received 100 CFU aerosolised Erdman K01 21 weeks after primary vaccination. | WB and PBMC | 0.3 ml WB or 1-3x10^6^ PBMCs plus autologous serum matched to animal and time point | BCG Pasteur or H37Rv; 500 CFU | 0.6 ml; 96 hours | Quantification of BCG in the lymph node | Mycobacterial growth in the direct WB MGIA inhibited following BCG vaccination and correlates with BCG recovered from the lymph node following *in vivo* BCG challenge; MGIA vaccine response correlated with total pathology, lung pathology, extrathoracic pathology and CRP |
| High-dose Mycobacterium tuberculosis aerosol challenge cannot overcome BCG-induced protection in chinese origin cynomolgus macaques; implications of natural resistance for vaccine evaluation [71] | 2021 | *Scientific Reports* | Sibley, L., White, A.D., Gooch, K.E., Stevens, L.M., Tanner, R., Jacobs, A., Daykin-Pont, O., Gleeson, F., McIntyre, A., Basaraba, R. and Clark, S. | NHP | Cynomolgus macaques | 12 | Six macaques (Group A) were immunised intradermally (ID) in the upper left arm with 100 μl BCG vaccine, Danish strain 1331 (SSI, Copenhagen, Denmark), and 6 were unvaccinated controls (Group B). Twenty-one weeks after vaccination, all animals were challenged by the aerosol route with *M. tb.* | PBMC | 3x10^6^ PBMCs plus autologous serum matched to animal and time point | BCG Pasteur ~500 CFU | 0.6 ml; 96 hours | Disease progression post-challenge, specific IFN-γ response, polyfunctional and memory T cell analysis, IgG titre | Not clear for immune parameters; for protection, no correlations with MGIA other than a trend towards associated with X-ray score |
| A mycobacterial growth inhibition assay (MGIA) for bovine TB vaccine development [75] | 2017 | *Tuberculosis* | Pepponi, I. and Khatri, B. and Tanner, R. and Villarreal-Ramos, B. and Vordermeier, M. and McShane, H. | Cow | Holstein-Friesian male cattle (approximately 6 months of age at study start) | 24 | 1x10^6^ CFU BCG Danish SSI | WB and PBMC | 0.3 ml WB or 1x10^6^ PBMCs collected at 2, 6-, 9-, 12- and 14-weeks following vaccination | BCG Pasteur; 3.5x10^3^ CFU | 0.6 ml; 96 hours | Flow cytometric analysis of PBMC immune populations: T lymphocytes, monocytes, γδ T cells and natural killer cells | Significant correlation found only between the proportion of NK T-like cells and level of growth inhibition |
| Functional *in-vitro* evaluation of the non-specific effects of BCG vaccination in a randomised controlled clinical study [79] | 2022 | *Scientific reports* | Wilkie, M. and Tanner, R. and Wright, D. and Lopez Ramon, R. and Beglov, J. and Riste, M. and Marshall, J. L. and Harris, S. A. and Bettencourt, P. J. G. and Hamidi, A. and van Diemen, P. M. and Moss, P. and Satti, I. and Wyllie, D. and McShane, H. | Human | Healthy BCG-naïve, UK adults | 35 | i.d. vaccination BCG SSI (2-8x10^5 CFU) or unvaccinated | PBMC | 3x10^6^ PBMC plus autologous serum | BCG Pasteur; 500 CFU | 0.6 ml; 96 hours | Ex-vivo IFNγ ELISpot; WB and PBMC growth inhibition assays (*Staphylococcus aureus, Escherichia coli, Klebsiella pneumoniae* and Streptococcus agalactiae) | No |
| A novel view on the pathogenesis of complications after intravesical BCG for bladder cancer [81] | 2018 | *International Journal of Infectious Diseases* | Bilsen, M. P. and van Meijgaarden, K. E. and de Jong, H. K. and Joosten, S. A. and Prins, C. and Kroft, L. J. M. and Jonker, J. T. and Crobach, S. and Pelger, R. C. and Ottenhoff, T. H. M. and Arend, S. M. | Human | Bladder cancer patients | 2 | BCG instillation | PBMC | 1x10^6^ PBMC | BCG; 100 CFU | Not specified; 96 hours | Proliferation of T-lymphocytes in response to M. tb PPD; levels of IFNγ production by peripheral blood mononuclear cells in response to PPD | No |
| Efficacy of tuberculosis vaccine candidate pcDNA3.1-rpfB in inhibiting the growth of mycobacterium tuberculosis *in vitro* with mycobacterial growth inhibition assay [82] | 2022 | *Makara Journal of Science* | Pujilestari, R. and Rukmana, A. and Karuniawati, A. | Mouse | 6–8-week-old BALB/c mice | Not specified | 100 ug/100 ul TB vaccine candidate pcDNA3.1-rpfB i.m., 100 ug/100 ul pcDNA3.1 i.m. or 100 µlBCG SSI i.p.. After the first injection, a booster was performed on days 14 and 28. | PBMC plus autologous serum and splenocytes | 3x10^6^ PBMC and splenocytes | H37Rv; 0.0005 McFarland | 0.6 ml; 96 hours | IFNγ ELISA | No correlation between the time to positivity values and IFNγ levels in the splenocyte or PBMC MGIA |
| Evaluation of tuberculosis vaccine candidate, pcDNA3.1-rpfD using mycobacterial growth inhibition assay (MGIA) [83] | 2022 | *HAYATI Journal of Biosciences* | Nurfadilah, M. and Rukmana, A. and Sjatha, F. | Mouse | 6–8-week-old BALB/c mice | 18 | Vaccination three times within two weeks interval with 100 μg/100 μl pcDNA3.1-rpfD i.m., pcDNA3.1 or 2-8x10^5^ CFU BCG SSI i.p. | PBMC and splenocytes | 3x10^6^ PBMC and splenocytes | H37Rv; 0.0005 McFarland | 0.6 ml; 96 hours | IFNγ ELISA | No correlation between IFNγ levels and time to positivity |
| The influence of haemoglobin and iron on *in vitro* mycobacterial growth inhibition assays [84] | 2017 | *Scientific reports* | Tanner, R. and O'Shea, M. K. and White, A. D. and Muller, J. and Harrington-Kandt, R. and Matsumiya, M. and Dennis, M. J. and Parizotto, E. A. and Harris, S. and Stylianou, E. and Naranbhai, V. and Bettencourt, P. and Drakesmith, H. and Sharpe, S. and Fletcher, H. A. and McShane, H. | Human and NHP | Five cohorts: i) healthy adults with or without a history of BCG vaccination, no evidence of latent M. tb infection; ii) healthy adults with or without a history of BCG vaccination, no past history of TB or evidence of latent infection; iii) healthy South African infants, aged 4–6 months), received BCG vaccination within seven days of birth; iv) rhesus and cynomolgus macaques; v) mouse | 12 + 21 + 128 +14 + 6 | Cohort i - single i.d. dose of 2-8x10^5 CFU BCG SSI; cohort iv - vaccinated i.d. in the upper left arm with BCG Danish strain 133, 100 μl of 0.2-0.8x10^6^ CFU | WB, PBMCs and murine splenocytes | 0.3 ml WB or 1x10^6^ PBMCs / mouse splenocytes +/- haemoglobin/ferric iron/deferoxamine; cohort i - 4-, 8- and 24-weeks post-vaccination; cohorts ii and iii) enrolment | BCG Pasteur Aeras, H37Rv; 150 CFU | 0.6 ml; 96 hours | Environmental and demographic parameters assessed for their effect on predicting mycobacterial growth: eosinophil count, time delay, bovine PPD response, avian PPD response, TLR6 diversity and the interaction of age group:sex | Eosinophil count, time delay, bovine PPD response and avian PPD response negatively correlated with mycobacterial growth; TLR6 diversity and age group in males positively correlated with mycobacterial growth |
| Safety and immunogenicity of the recombinant BCG Vaccine AERAS-422 in healthy BCG-naive adults: a randomized, active-controlled, first-in-human phase 1 trial [85] | 2016 | *EBioMedicine* | Hoft, D. F. and Blazevic, A. and Selimovic, A. and Turan, A. and Tennant, J. and Abate, G. and Fulkerson, J. and Zak, D. E. and Walker, R. and McClain, B. and Sadoff, J. and Scott, J. and Shepherd, B. and Ishmukhamedov, J. and Hokey, D. A. and Dheenadhayalan, V. and Shankar, S. and Amon, L. and Navarro, G. and Podyminogin, R. and Aderem, A. and Barker, L. and Brennan, M. and Wallis, R. S. and Gershon, A. A. and Gershon, M. D. and Steinberg, S. | Human | Healthy HIV-negative, BCG-naïve, mycobacteria-naïve individuals | 24 | I.d. immunisation with low-dose (1x10^5^-1x10^6^ CFU), high-dose (1x10^5^-1x10^6^ CFU) AERAS-422 or Tice BCG | WB obtained at 3-, 7-, 14-, 28-, 56-, 84-, 112-, 140- and 182-days post-vaccination | 0.3 ml WB | 150 CFU BCG Tice | 0.6 ml; 96 hours | WB lymphoproliferative assay, blood transcriptome and ICS | No difference in IFNγ responses post-vaccination between vaccine groups detected after stimulation with overexpressed recombinant antigens or BCG; no difference in inflammatory cytokine responses to infection with BCG or Aeras-422; across all vaccine groups and time points, positive correlations observed between both IL-1β and a monocyte chemokine module and delta WBA. |
| Safety and immunogenicity of a thermostable ID93 + GLA-SE tuberculosis vaccine candidate in healthy adults [86] | 2023 | *Nature Communications* | Sagawa, Z. K. and Goman, C. and Frevol, A. and Blazevic, A. and Tennant, J. and Fisher, B. and Day, T. and Jackson, S. and Lemiale, F. and Toussaint, L. and Kalisz, I. and Jiang, J. and Ondrejcek, L. and Mohamath, R. and Vergara, J. and Lew, A. and Beckmann, A. M. and Casper, C. and Hoft, D. F. and Fox, C. B. | Human | Healthy BCG-naïve adults | 48 | i.m. vaccination with ID93 + GLA-SE on study days 0 and 56 | WB | 0.3 ml WB obtained on study days 0, 7 and 224 | BCG; inoculum not specified | 0.6 ml; 72 hours | Serum ELISAs; secretory IgA ELISAs on nasal swabs and tear samples; intracellular cytokine staining and flow cytometry; short- and long-term B cell, and IFNγ and IL-10 T cell ELISpot assays | No |
| RUTI vaccination enhances inhibition of mycobacterial growth *ex vivo* and induces a shift of monocyte phenotype in mice [87] | 2019 | *Frontiers in Immunology* | Prabowo, S. A. and Painter, H. and Zelmer, A. and Smith, S. G. and Seifert, K. and Amat, M. and Cardona, P. J. and Fletcher, H. A. | Mouse | 6–8-week-old female C57BL/6 mice | 6 mice/group | s.c. vaccination with BCG or 204 ug RUTI with specific groups receiving a second vaccination three weeks later | Splenocytes | 5x10^6^ splenocytes collected at 1-, 3-, 4-, 6- and 9-weeks post vaccination | BCG Pasteur; 90 CFU | 0.6 ml; 96 hours | IFNγ ELISpot response to PPD; cell surface staining and flow cytometry; qRT-PCR | Induction of IFNγ -secretion did not correlate with ability to control mycobacterial growth; non-significant correlation between higher frequency of Ly6C^-^ monocytes/macrophages following RUTI vaccination and lower growth of mycobacteria across time course |
| Immunological correlates of mycobacterial growth inhibition describe a spectrum of tuberculosis infection [88] | 2018 | *Scientific reports* | O'Shea, M. K. and Tanner, R. and Muller, J. and Harris, S. A. and Wright, D. and Stockdale, L. and Stylianou, E. and Satti, I. and Smith, S. G. and Dunbar, J. and Fletcher, T. E. and Dedicoat, M. and Cunningham, A. F. and McShane, H. | Human | HIV-negative adults with suspected or microbiologically confirmed pulmonary or extrapulmonary TB disease, latent TB infection and health uninfected controls | 171 | Treatment for active disease and LTBI; drug regimen not specified | WB | 0.3 ml WB pre-treatment and 1-6 months post-treatment | H37Rv or BCG; volume predicted to be positive in 6.5 days | 0.6 ml; 96 hours | Immunophenotyping of monocytes and B cells, anti-M. tb IgG isotype ELISAs and multiplex serum cytokine and chemokine analysis | Mycobacterial control correlated with inflammatory monocytes, activated and atypical memory B cells, IgG1 responses to TB-specific antigens and serum cytokines/chemokines |
| Disparate Tuberculosis Disease Development in Macaque Species Is Associated With Innate Immunity [89] | 2019 | *Frontiers in immunology* | Dijkman, K., and Vervenne, R.A., and Sombroek, C.C., and Boot, C., and Hofman, S.O., and Van Meijgaarden, K.E., and Ottenhoff, T.H., and Kocken, C.H., and Haanstra, K.G., and Vierboom, M.P. and Verreck, F.A. | NHP | Ten male, non-Mauritian cynomolgus macaques (Macaca fascicularis) and 10 male Indian-type rhesus macaques (Macaca mulatta) | 20 | Animals were challenged with increasing doses of Mycobacterium tuberculosis Erdman K01 strain (BEI Resource, VA, USA). Each Mtb challenge dose was delivered by endobronchial instillation of 3 mL inoculum, targeting the lower left lung lobe | PBMC | 1x10^6^ PBMC | 100 CFU BCG Pasteur | 0.6 ml; 96 hours | Post-challenge pathology, bacterial load in tissue, flow cytometric analysis of local and peripheral immune subsets, antibody ELISAs, multiplex cytokine assay | Reduced pathological involvement and bacterial tissue burden observed in cynomolgus compared to rhesus macaques was not reflected in differential bacterial outgrowth control as measured by PBMC-based MGIA |
| Application of a whole blood mycobacterial growth inhibition assay to study immunity against *Mycobacterium tuberculosis* in a high tuberculosis burden population [90] | 2017 | *PLoS ONE* | Baguma, R. and Penn-Nicholson, A. and Smit, E. and Erasmus, M. and Day, J. and Makhethe, L. and De Kock, M. and Hughes, E. J. and Van Rooyen, M. and Pienaar, B. and Stone, L. and Hanekom, W. and Brennan, M. J. and Wallis, R. S. and Hatherill, M. and Scriba, T. J. | Human | Three cohorts of BCG-vaccinated HIV-negative participants from Western Cape; cohort 1 - 19-51 year-old adults, cohort 2 - 18-year-old adults, cohort 3 - 8-year-old children; equal numbers of M. tb infected and uninfected participants in each cohort | 161 | None | WB | 0.3 ml WB | BCG Danish, HN878, H37Rv, CDC1551; volume predicted to be positive in 6.5 days | 0.6 ml; 96 hours | WB ICS | Infection status, age of the study participants or strain was not associated with differential control of mycobacterial growth; abundance and function of innate or T-cell responses were not associated with mycobacterial growth |
| Comparison of two mycobacterial strains in performance of the whole blood mycobacterial growth inhibition assay in Indian children [91] | 2022 | *Tuberculosis* | Venkataraman, A. and Shanmugam, S. and Balaji, S. and Mani, K. and Shanmugavel, A. K. and Muthuramalingam, K. and Hissar, S. and Thiruvengadam, K. and Selladurai, E. and Smuk, M. and Hanna, L. E. and Prendergast, A. J. | Human | <5-year-old children who were healthy household contacts of adults with pulmonary tuberculosis | 30 | None | WB | 0.3 ml WB | BCG or H37Rv; 250 (BCG) or 720 (H37Rv) CFU | 0.6 ml; 72 hours | No | NR |
| Human hookworm infection enhances mycobacterial growth inhibition and associates with reduced risk of tuberculosis infection [98] | 2018 | *Frontiers in Immunology* | O'Shea, M. K. and Fletcher, T. E. and Muller, J. and Tanner, R. and Matsumiya, M. and Bailey, J. W. and Jones, J. and Smith, S. G. and Koh, G. and Horsnell, W. G. and Beeching, N. J. and Dunbar, J. and Wilson, D. and Cunningham, A. F. and McShane, H. | Human | Hookworm infected male Nepalese military recruits recently arrived in UK and matched healthy controls | 34 | Hookworm treatment, drug regimen not specified | WB and PBMC | 0.3 ml WB or 3x10^6^ PBMCs obtained pre- and post-treatment, time frame not specified | H37Rv; 150 CFU (WB) or 10 CFU (PBMCs) | 0.6 ml; 96 hours | Anti-M. tb IgG1 ELISA and WB microarrays | Significant negative relationship between mycobacterial growth and eosinophil counts. Eosinophil-associated differential gene expression characterised the WB transcriptome of hookworm infection and correlated with improved mycobacterial control |
| Mycobacterial growth inhibition assay (MGIA) as a host directed diagnostic tool for the evaluation of the immune response in subjects living with type 2 diabetes mellitus [99] | 2021 | *Frontiers in Cellular and Infection Microbiology* | Bobadilla-del-Valle, M. and Leal-Vega, F. and Torres-Gonzalez, P. and Ordaz-Vazquez, A. and Garcia-Garcia, M. D. L. and Tovar-Vargas, M. D. L. A. and Delgado-Sanchez, G. and Guerra De Blas, P. D. C. and Wallis, R. S. and Ponce-De-Leon, A. and Sifuentes-Osornio, J. | Human | Adults with type-2 diabetes with optimal (HbA1c ≤ 7%) or poor glycaemic control (HbA1c ≥ 8%), or healthy controls | 54 + 35 + 44 | None | WB | 0.3 ml WB | H37Rv or BCG; volume of predicted to be positive in 7 days | 0.6 ml; 96 hours | Cytometric bead array (human T_H_1/T_H_2/T_H_17) | No difference in capacity to control mycobacterial growth between healthy subjects and patients with optimal glycaemic control, significant differences between healthy subjects and patients with poor glycaemic control; no differences in IL-2, IL-4 and IL-17A levels between healthy subjects and patients with poor control, significant decrease in the level of IL-6, IL10 and TNFα in patients with poor glycaemic control versus healthy controls |
| Glibenclamide reduces primary human monocyte functions against tuberculosis infection by enhancing M2 polarization [100] | 2018 | *Frontiers in Immunology* | Kewcharoenwong, C. and Prabowo, S. A. and Bancroft, G. J. and Fletcher, H. A. and Lertmemongkolchai, G. | Human | Healthy UK individuals; diabetic Thai individuals; Thai individuals | 41 (UK) + 15 (Thailand) | None | Monocytes | Number of monocytes used not specified + 50, 100 μM glibenclamide | BCG Pasteur Aeras or H37Rv; 100 CFU | 0.6 ml; 96 hours | Monocyte stimulation and cytokine analysis by ELISA; cell surface marker staining and analysis by flow cytometry | Consistent with reduced mycobacterial control in monocyte samples pre-treated with glibenclamide, reduced M1 surface markers and TNFα production, and enhanced M2 surface markers and IL-10 production were reported |
| Distinct transcriptional and anti-mycobacterial profiles of peripheral blood monocytes dependent on the ratio of monocytes: lymphocytes [101] | 2015 | *EBioMedicine* | Naranbhai, V. and Fletcher, H. A. and Tanner, R. and O'Shea, M. K. and McShane, H. and Fairfax, B. P. and Knight, J. C. and Hill, A. V. | Human | Healthy adult IGRA-negative BCG-vaccinated Caucasian volunteers | 29 (WB) + 13 (mixed leukocyte) | None | WB or mixed leucocyte | 0.3 ml WB or 1x10^6^ mixed leukocytes (monocytes and non-monocytes) | BCG Pasteur; inoculum not specified | Volume not specified; 96 hours (WB), 72 hours (mixed leukocytes) | *In vivo* and *in vitro* ML ratio; transcriptional profiling by microarray of CD14^+^ monocytes | *In vivo* but not *in vitro* ML ratio significantly associated with mycobacterial growth |
| Toll-like receptor (TLR) diversity influences mycobacterial growth in African buffalo [102] | 2017 | *Tuberculosis* | le Roex, N. and Jolles, A. and Beechler, B. and van Helden, P. and Hoal, E. | African Buffalo | Buffalo from Kruger National Park where Bovine TB is widespread | 43 | None | WB | 0.3 ml WB | BCG Danish 1331; inoculum not specified | 0.6 ml; 96 hours |  |  |
| Association of human antibodies to arabinomannan with enhanced mycobacterial opsonophagocytosis and intracellular growth reduction [105] | 2016 | *Journal of Infectious Diseases* | Chen, T. and Blanc, C. and Eder, A. Z. and Prados-Rosales, R. and Souza, A. C. O. and Kim, R. S. and Glatman-Freedman, A. and Joe, M. and Bai, Y. and Lowary, T. L. and Tanner, R. and Brennan, M. J. and Fletcher, H. A. and McShane, H. and Casadevall, A. and Achkar, J. M. | Human | Healthy, M. tb-uninfected adults | 17 | Primary or secondary i.d. immunisation with BCG SSI | PBMCs obtained 4-, 8- and 24-weeks post vaccination | 1x10^6^ PBMCs | 600 CFU BCG Pasteur | 0.6 ml; 96 hours | IgG titres to arabinomannan at four-weeks post vaccination | Mycobacterial growth inhibition showed a significant correlation with IgG responses to arabinomannan four weeks post vaccination |
| Impact of selective immune-cell depletion on growth of *Mycobacterium tuberculosis* (Mtb) in a whole-blood bactericidal activity (WBA) assay [107] | 2019 | *PLoS ONE* | Cross, G. B. and Yeo, B. C. M. and Hutchinson, P. E. and Tan, M. C. and Verma, R. and Lu, Q. and Paton, N. I. | Human | Healthy volunteers | 8 | None | WB, WB depleted of neutrophils, dendritic cells, monocytes, NK cells, CD4^+^ or CD8^+^ T cells, or B cells | 0.3 ml WB | H37Rv; volume predicted to be positive in 5.5 days | 0.6 ml; 72 hours +/- 1 μg/ml rifampicin | IGRA | No significant difference in growth in any WBA experimental conditions comparing results for IGRA-positive or -negative volunteers. |
| Regulation of mycobacterial infection by macrophage Gch1 and tetrahydrobiopterin [111] | 2018 | *Nature Communications* | McNeill, E. and Stylianou, E. and Crabtree, M. J. and Harrington-Kandt, R. and Kolb, A. L. and Diotallevi, M. and Hale, A. B. and Bettencourt, P. and Tanner, R. and O'Shea, M. K. and Matsumiya, M. and Lockstone, H. and Muller, J. and Fletcher, H. A. and Greaves, D. R. and McShane, H. and Channon, K. M. | Human and mouse | Human: Healthy South African infants who received BCG within seven days of birth; mouse: <10-week-old Gch1 ^fl/fl^Tie2^cre^, Gch1 ^fl/fl^, Nos2^-/-^ and C57BL/6J female mice | 78 human participants and 5-7 mice per group | None | Human PBMCs and mouse BMDMs | 1x10^6^ human PBMCs; mouse BMDM input not specified | BCG Pasteur; human MGIA 600 CFU; mouse MGIA MOI 50:1 | 0.6 ml; 96 hours +/- IFNγ (mouse MGIA only) | Gene expression analysis of GCH1 and NOS2 | Decreased Gch1 expression correlates with enhanced cell-intrinsic control of mycobacterial infection *in vitro* |

**Supplementary Table 2. Data collected from included studies by order of citation in results.** BCG, Bacillus Calmette–Guérin; BALCs, bronchoalveolar lavage cells; CFU, colony forming units; ELISA, enzyme-linked immunosorbent assay; ELISpot, enzyme-linked immunosorbent spot; ICS, intracellular cytokine staining; i.d., intradermal; IFN. Interferon; IGRA. Interferon-gamma release assay; IL, interleukin; i.p. intraperitoneal; i.m., intramuscular; i.t., intratracheal; i.v., intravenous; LTBI, latent tuberculosis infected; MDR, multidrug-resistant; M. tb, *Mycobacterium tuberculosis*; NHP, Non-human primate; NR, not relevant; PBMC, peripheral blood mononuclear cells; PPD, purified protein derivative; QFT, QuantiFERON; RFU, relative fluorescence unit; s.c., subcutaneous; TB, tuberculosis; TNF, tumour necrosis factor; TTP, time to positivity; WB, whole blood.

| **Publication and reference number** | **Objective** | **Population** | **Sample size** | **Intervention** | **Randomisation** | **Controls** | **Timeframe** | **Blinding** | **Replicates** | **Methods** | **Outcome**  **measures** | **Statistical**  **analysis** | **Quality score** | **Quality**  **category** |
| --- | --- | --- | --- | --- | --- | --- | --- | --- | --- | --- | --- | --- | --- | --- |
| Wallis et al. 2001 [16] | Y | N | N | Y | NA | Y | Y | NR | NR | Y | Y | Y | 5 | Fair |
| Wallis et al. 2003 [22] | Y | Y | Y | Y | NA | Y | Y | NA | NR | Y | Y | Y | 9 | Very good |
| Janulionis et al. 2005 [23] | Y | Y | N | NA | NA | NA | NA | NA | Y | Y | Y | N | 3 | Fair |
| Wallis et al. 2009 [24] | Y | Y | Y | NA | NA | Y | NA | NA | N | N | Y | Y | 4 | Fair |
| Wallis et al. 2022 [25] | Y | Y | Y | Y | Y | Y | Y | Y | NR | N | Y | Y | 9 | Very good |
| Janulionis et al. 2004 [26] | Y | Y | N | Y | NR | Y | Y | NR | NR | N | Y | NR | 4 | Fair |
| Wallis et al. 2010 [27] | Y | Y | Y | Y | NR | Y | Y | Y | Y | N | Y | Y | 9 | Very good |
| Wallis et al. 2014 [28] | Y | Y | Y | Y | Y | Y | Y | Y | NR | N | Y | Y | 9 | Very good |
| Wallis et al. 2018 [29] | Y | Y | Y | Y | Y | Y | Y | N | Y | N | Y | Y | 8 | Good |
| Wallis et al. 2011 [30] | Y | Y | Y | Y | NR | Y | Y | NR | NR | N | Y | Y | 7 | Good |
| Gurumurthy et al. 2017 [31] | Y | Y | Y | Y | Y | Y | Y | Y | Y | N | Y | Y | 10 | Very good |
| Zhu et al. 2014 [32] | Y | Y | Y | Y | NR | Y | Y | NR | NR | N | Y | Y | 7 | Good |
| Naftalin et al. 2017 [33] | Y | Y | Y | Y | Y | Y | Y | NR | Y | N | Y | Y | 9 | Very good |
| Naftalin et al. 2018 [34] | Y | Y | Y | Y | Y | Y | Y | NR | Y | N | Y | Y | 9 | Very good |
| Verma et al. 2022 [35] | Y | N | Y | Y | NA | N | Y | NR | Y | Y | Y | Y | 6 | Good |
| Saliu et al. 2006 [36] | Y | Y | Y | NA | NA | Y | Y | NA | NR | N | Y | Y | 6 | Good |
| Harausz et al. 2016 [37] | Y | Y | Y | NA | NA | Y | Y | NA | NR | Y | Y | Y | 8 | Good |
| Wallis et al. 2012 [38] | Y | Y | N | NA | NA | Y | NA | NR | Y | N | Y | Y | 4 | Fair |
| Kwan et al. 2020 [39] | Y | Y | N | NA | NA | Y | Y | NA | Y | Y | Y | N | 5 | Fair |
| Reddy et al. 2012 [40] | Y | N | N | NA | NA | Y | Y | NA | Y | N | Y | Y | 3 | Fair |
| Wallis et al. 2004 [41] | NA | NA | NA | NA | NA | NA | NA | NA | NA | NA | NA | NA | NA | NA |
| Cheon et al. 2002 [42] | Y | Y | Y | Y | NA | Y | Y | NR | Y | Y | Y | Y | 10 | Very good |
| Fletcher et al. 2013 [43] | Y | Y | Y | Y | NA | Y | Y | NR | Y | Y | Y | Y | 10 | Very good |
| Lee et al. 2019 [45] | Y | Y | Y | NA | NA | Y | NA | NA | Y | Y | Y | N | 6 | Good |
| Smith et al. 2016 [46] | Y | Y | Y | Y | NA | Y | NR | Y | Y | Y | Y | Y | 10 | Very good |
| Joosten et al. 2018 [47] | Y | Y | Y | Y | NR | Y | Y | Y | Y | Y | Y | Y | 11 | Excellent |
| Prabowo et al. 2019 [48] | Y | Y | Y | NA | NA | Y | NA | NA | NR | Y | Y | Y | 7 | Good |
| Proabowo et al. 2019 [49] | Y | Y | Y | NA | NA | Y | Y | NA | NR | Y | Y | Y | 8 | Good |
| Tanner et al. 2019 [50] | Y | N | N | Y | NR | Y | Y | NR | Y | Y | Y | Y | 6 | Good |
| Tanner et al. 2021 [51] | Y | N | Y | NA | NR | N | NA | N | Y | Y | Y | Y | 3 | Fair |
| Bitencourt et al. 2021 [52] | Y | Y | Y | Y | Y | Y | Y | NR | N | Y | Y | Y | 9 | Very good |
| Harris et al. 2014 [53] | Y | Y | Y | Y | NR | Y | Y | NR | NR | N | Y | Y | 7 | Good |
| Tanner et al. 2020 [56] | Y | Y | Y | Y | Y | Y | Y | NR | N | Y | Y | Y | 9 | Very good |
| Radloff et al. 2018 [61] | Y | Y | Y | NA | NA | Y | Y | NA | Y | Y | Y | Y | 9 | Very good |
| Andersson et al. 2020 [62] | Y | N | Y | NA | NA | Y | NA | NA | Y | N | Y | Y | 4 | Fair |
| Marsay et al. 2013 [63] | Y | Y | Y | Y | NR | Y | Y | NR | Y | N | Y | Y | 8 | Good |
| Zelmer et al. 2016 [64] | Y | Y | N | Y | NR | Y | Y | NR | NR | Y | Y | Y | 7 | Good |
| Yang et al. 2016 [65] | Y | Y | N | Y | NR | Y | Y | NR | Y | Y | Y | Y | 8 | Good |
| Jensen et al. 2017 [66] | Y | Y | N | Y | NR | Y | N | NR | Y | Y | Y | N | 4 | Fair |
| Painter et al. 2020 [67] | Y | Y | N | Y | Y | Y | Y | NR | Y | Y | Y | Y | 9 | Very good |
| Tanner et al. 2021 [70] | Y | Y | Y | Y | Y | Y | Y | N | Y | Y | Y | Y | 10 | Very good |
| Sibley et al. 2021 [71] | Y | Y | Y | Y | Y | Y | Y | N | NR | Y | Y | N | 7 | Good |
| Pepponi et al. 2017 [75] | Y | Y | Y | Y | NR | Y | Y | NR | Y | Y | Y | Y | 10 | Very good |
| Wilkie et al. 2022 [79] | Y | Y | Y | Y | Y | Y | Y | Y | Y | Y | Y | Y | 12 | Excellent |
| Bilsen et al. 2018 [81] | N | Y | N | N | NA | Y | Y | NA | Y | N | Y | NA | 1 | Poor |
| Pujilestari et al. 2022 [82] | Y | Y | NR | Y | NR | N | N | NR | NR | Y | Y | N | 2 | Poor |
| Nurfadilah et al. 2022 [83] | Y | Y | NR | Y | NR | N | N | NR | NR | Y | Y | N | 2 | Poor |
| Tanner et al. 2017 [84] | Y | Y | Y | Y | NR | Y | Y | NR | Y | Y | Y | Y | 10 | Very good |
| Hoft et al. 2016 [85] | Y | Y | Y | Y | Y | Y | Y | Y | NR | Y | Y | Y | 11 | Excellent |
| Sagawa et al. 2023 [86] | Y | Y | Y | Y | Y | Y | Y | Y | NR | N | Y | Y | 9 | Very good |
| Prabowo et al. 2019 [87] | Y | Y | Y | Y | NR | Y | Y | NR | NR | Y | Y | Y | 9 | Very good |
| O’Shea et al. 2018 [88] | Y | Y | Y | Y | NA | Y | Y | NA | Y | Y | Y | Y | 10 | Very good |
| Dijkman et al. 2019 [89] | Y | Y | Y | Y | Y | Y | Y | Y | NR | Y | Y | N | 9 | Very good |
| Baguma et al. 2017 [90] | Y | Y | Y | Y | NA | NA | Y | NA | NA | Y | Y | Y | 8 | Good |
| Venkataraman et al. 2022 [91] | Y | Y | Y | NA | NA | N | NA | Y | Y | Y | Y | Y | 7 | Good |
| O’Shea et al. 2018 [98] | Y | Y | Y | Y | NA | Y | NR | NA | Y | Y | Y | Y | 9 | Very good |
| Bobadilla-del-Valle et al. 2021 [99] | Y | Y | Y | NA | NA | Y | NA | NA | Y | N | Y | Y | 6 | Good |
| Kewcharoenwong et al. 2018 [100] | Y | Y | N | NA | NA | N | NA | NA | Y | N | Y | Y | 2 | Poor |
| Naranbhai et al. 2015 [101] | Y | Y | Y | NA | NA | Y | NA | NA | NR | N | Y | Y | 5 | Fair |
| Le Roex et al. 2017 [102] | Y | Y | Y | NA | NA | NA | NA | NA | Y | N | Y | Y | 5 | Fair |
| Chen et al. 2016 [105] | Y | Y | Y | Y | NA | Y | Y | NR | Y | Y | Y | Y | 10 | Very good |
| Cross et al. 2019 [107] | Y | N | Y | NA | NA | Y | NA | NA | Y | N | Y | N | 2 | Poor |
| McNeill et al. 2018 [111] | Y | Y | N | NA | NA | Y | NA | NA | Y | N | Y | Y | 4 | Fair |

**Supplementary Table 3: Risk of bias (quality) assessment of included studies by order of citation in results. Strategy adapted from a standardized quality assessment tool developed by NHLBI and the QUIN Tool for relevance to the reported studies.**

*Possible responses: Y, N, NR (not reported), NA (not applicable); Quality category (0-2=poor, 3-5=fair, 6-8=good, 9-10=very good, 11-12=excellent)*

1. Is the study question or objective clearly stated?

2. Are the study population(s) pre-specified and clearly described?

3. Is the sample size sufficiently large to provide confidence in the findings?

4. Are any interventions clearly described and delivered consistently?

5. Where individuals are assigned to an intervention, is the method of randomization adequate (i.e., use of randomly generated assignment)?

6. Are appropriate control group(s) included?

7. Is the timeframe sufficient so that one could reasonably expect to see an association between intervention and outcome if it existed?

8. Were the people assessing the outcomes blinded to the participants' interventions?

9. Are appropriate replicates used?

10. Are the assay methods described in sufficient detail to allow replication?*

11. Are the outcome measures pre-specified, clearly defined, valid, reliable, and assessed consistently?

12. Is the appropriate statistical analysis applied and clearly specified? Are the presented results based on predefined aims and/or objectives, and is all data adequately tabulated with baseline data clearly specified (if applicable)?

*Methods were considered to be described in insufficient detail if the following key details were omitted: mycobacterial input inoculum in CFU, cell number, whole blood volume or total co-culture volume.
